# Activation peptide of the coagulation factor XIII (AP-F13A1) as a new biomarker for the screening of colorectal cancer

**DOI:** 10.1186/s12014-018-9191-3

**Published:** 2018-04-09

**Authors:** Julien Peltier, Jean-Pierre Roperch, Stéphane Audebert, Jean-Paul Borg, Luc Camoin

**Affiliations:** 1 Aix Marseille Univ, CNRS, INSERM, Institut Paoli-Calmettes, CRCM, Marseille Protéomique, Marseille, France, Aix Marseille Univ, 27 Bd Leï Roure, BP30059, 13273 Marseille Cedex 9, France; 2 PROFILOME SAS, 24 Rue du Faubourg Saint-Jacques, 75014 Paris, France; 3 Present Address: Institute for Cell and Molecular Biosciences, Faculty of Medical Sciences, Institute for Cell and Molecular Biosciences, Newcastle University, Newcastle Upon Tyne, NE2 4HH UK; 4 Present Address: OncoDiag, Pépinière scientifique, Rue de Pacy, Miserey, France

**Keywords:** Colorectal cancer, Biomarker, AP-F13A1, ELISA, LC-PRM

## Abstract

**Background:**

Colorectal cancer (CRC) remains a major cause of cancer fatalities in developed countries. The risk of death is correlated to the stage of CRC during the primary diagnosis. Early diagnosis is closely associated with enhanced survival rate. We therefore investigated the AP-F13A1 as a potential protein marker of CRC.

**Methods:**

The protein expression of FXIII in 40 serum samples was evaluated by enzyme-linked immunosorbent assays. Additionally, targeted proteomic assays (LC-PRM) were used to evaluate the expression of the activation peptide of F13A1 (AP-F13A1) in a further 113 serum samples. Results were analyzed by the Wilcoxon test and receiver operating characteristic curves generated to assess statistical differences and diagnostic factors between CRC patients and controls.

**Results:**

AP-F13A1 was quantified in human serum samples using calibration curves with excellent linearity. AP-F13A1 was reduced in CRC patients using PRM assays from two distinct biobanks. The AUC for AP-F13A1 were 0.95 and 0.93. Sensitivity/specificity values for the two sets of patients were 75%/95% and 71%/95% respectively.

**Conclusion:**

We have presented the proof of principle that in vivo release of AP-F13A1 can be measured by PRM-based strategies in CRC serum samples. AP-F13A1 may be an effective serological biomarker as part of a screening program of CRC detection.

**Electronic supplementary material:**

The online version of this article (10.1186/s12014-018-9191-3) contains supplementary material, which is available to authorized users.

## Background

Colorectal cancer (CRC) remains a major cause of cancer mortality throughout the world with an annual incidence of approximately 1 million cases and an annual mortality of around 700,000. Western lifestyle and the aging population are the major factors accounting for the increased incidence of CRC [1]. CRC outcome is highly dependent upon the stage of detection. The 5-year survival rate of patients with metastases (stage III + IV) is less than 30%, while patients with localized adenomatous polyps (Stage I + II) have an excellent outcome with a 80% 5-year survival rate [2]. Unfortunately, initial clinical symptoms often occur at the advanced stage of CRC, when the disease has already progressed and spread beyond the colon. Detecting CRC by iFOBT in average risk individuals, i.e. asymptomatic 50–75 years individuals, can impact survival [3]. The iFOBT which uses specific antibodies for the globin portion of human haemoglobin to screen for blood in stool samples, has an acceptable 95% specificity but poor sensitivity of detection in the asymptomatic population (27% sensitivity for adenoma, 65% sensitivity for carcinoma) [3]. Presently, there is no noninvasive alternative to the recommended screening FOBT test [4]. Discovery of new non invasive biomarkers with greater diagnostic power are required to develop a more sensitive, reliable and non-invasive test for all forms of CRC [5].

A biomarker is defined as a biological indicator of the physiology and the pathological condition of patients and can be characterized by analysing blood milieu. We assume that comparison of blood samples from patients with adenomatous polyps or CRC will contain a molecular signature resulting from tissue damage or tumor production that will be different from the blood of healthy patients. In clinical laboratories, ELISA or other immunoassays are often the “gold standard” method to detect and quantify the protein expression with high throughput analysis with and an outstanding sensitivity. However, the limited specificity of many ELISAs and the cost for development put limitations on blood biomarkers [6]. An alternative approach based on the absolute quantification of molecules using mass spectrometry and single reaction monitoring (SRM) or parallel reaction monitoring (PRM) has been successfully implemented in clinical assays for monitoring drugs, metabolites and more recently proteins and peptides [7–9]. The SRM/PRM methods have the advantage of rapid and high specificity of detection for monitoring both the intact peptide mass and their fragments during LC–MS analysis.

Our group reported recently an iTRAQ-based proteomic strategy in different clinical stages of CRC [10]. In that discovery study, we selected a panel of potential biomarkers for evaluation on individual patients. One particular protein of interest, the coagulation factor XIII-A (F13A1) was identified as one of the key down-regulated proteins. In the present study, we develop and describe the outstanding potential of LC–MS/MS and PRM assays for quantifying and validating the endogenous F13A1 in cohorts of human serum samples at different stage of colorectal cancer. AP-F13A1 was found as a molecular biomarker that decreased in the serum of the CRC patients indicating that it may help in pre-selecting patients for colonoscopy.

## Methods

### Study subjects and sample collections

Human serum samples were taken from two biobank collections CCR1 (collection registered under 004-02 of CCP Paris-Est) and Valihybritest (ANR-10-BIOT-0012). To be eligible for inclusion, the patients had to have no previous history of colon or rectal surgery, of diseases such as cancer, inflammatory or infectious disease of the intestine, and no previous emergency colonoscopy. In all cases, serum samples were collected prior to colonoscopy. The signature of a consent form, approved by the ethical committee of Val de Marne Paris-Est area, authorized the recruitment of patients in all associated centers. Any special diets and medications during this period were recorded. The study continued until colonoscopy and pathology data had been checked. The final status was assigned as “normal colonoscopy (N)” and “cancer at colonoscopy (C)”. Samples used in our study were summarized in Table 1.

**Table 1:** Clinico-pathological data

	Biobank 1		Biobank 2	
Clinical features	Control patients (N)	Invasive cancer (C)	Control patients (N)	Invasive cancer (C)
Gender				
Female	12 (57%)	9 (47%)	24 (52%)	16 (59%)
Male	9 (43%)	10 (53%)	22 (48%)	11 (41%)
Age (years, Median [range])	52 [19–64]	69 [51–87]	52 [23–81]	66 [27–90]
Weight (kg, Median [range])	70 [48–115]	70 [50–100]	71 [42–130]	65 [40–89]
Size (cm, Median [range])	164 [155–193]	168 [157–182]	168 [145–187]	165 [153–185]
Cancer stage				
I		2 (11%)		4 (15%)
II		5 (26%)		10 (37%)
III		5 (26%)		7 (26%)
IV		7 (37%)		6 (22%)
Tumor location				
Right colon		11 (60%)		N/A
Left colon		8 (40%)		N/A

Patients were selected randomly from two collections and included for the validation of FXIII by ELISA and AP-F13A1 by PRM assays. For cancer patients, they were classified according to cancer’s stage (I, II, III, IV) and the location of the primary tumor

### Identification of F13A1 peptides

#### Solid phase extraction-purification

200 μL of delipidated serum samples [11] were diluted 5 times in 0.1% TFA prior to C18 solid phase extraction (SPE). The SPE cartridges (PID# WAT020515, Waters) were washed stepwise in 90% ACN and finally equilibrated 3 times in 0.1% TFA before the loading of diluted serum. Then, the SPE cartridge was washed three times with 0.1% TFA to remove binding of non-specific proteins on the C18 reverse phase and elution carried out with 70% ACN, 0.1% TFA.

#### Identification by mass spectrometry

Identifications were carried out on high resolution mass spectrometers from Thermo Scientific. The instrument method of the Q-Exactive was set-up as follows: Peptide fragments were measured in a survey full scan acquired in the Orbitrap in the range of m/z 400–5000 at a FWHM resolution of 140,000 at 200 m/z. The 12 abundant precursor ions were selected and HCD fragmentation was performed at 25% collision energy. Fragments were ejected from the HCD cell and read out in the Orbitrap mass analyser at a FWHM resolution of 17,500. Raw files generated from mass spectrometry analysis were processed with Proteome Discoverer 1.3 (Thermo Fisher Scientific). Proteome discoverer was used to search data via Mascot against the Human database subset of the Uniprot database (version 2013.06) containing 122 191 entries. Database searches were done using the following settings: no enzyme as cleavage, and acetyl N-terminal as variable modification. Mass tolerance of 6 ppm and 0.1 Da were used respectively for precursor and fragment ions during search analysis.

### ELISA assays

21 sera from healthy patients (N) and 19 from CRC patients (C) were extracted from the sample collection (Table 1; Biobank 1) and used for the quantification of the F13A1 polypeptide, using two commercially available ELISA kits according to the manufacturer’s instructions PID #ab108836 (Abcam) and PID #E91094Hu (USCN).

### PRM assays

#### Samples preparation

Serum samples were prepared as mentioned in 2.2.1 using the SPE purification. Samples were dried down under vacuum and digested overnight by adding 10 µg of trypsin (Promega). The pH was checked to ensure a complete digestion. A second SPE was performed using the process previously described above. Samples were dried under vacuum and stored at − 80 °C. The overall workflow of the study from biomarker discovery to the validation by LC-PRM is given in Additional file 1.

#### Optimisation of AP-F13A1 targets (tAP-F13A1)

LC–MS experiments were first performed on a LTQ Orbitrap Velos to confirm the detection of AP-F13A1 variants in SPE simplified serum. These experiments were accomplished by the use of an inclusion list in the mass spectrometry instrument method. Our inclusion list consisted of the precursor mass (m/z), charge (z), and collision energy for the tryptic peptides produced from the tAP-F13A1 (Additional file 2a). The values included in the list were provided by the following rules; (1) the peptide is fully tryptic and does not contain missed cleavage, (2) the m/z value of the peptide is between 400 and 1500 at a given charge, (3) no modifications are considered, except the intentional modifications performed on the heavy synthetic peptides. Instrument method was set up in data dependant mode to switch consistently between MS and MS/MS. No signal threshold for an MS/MS event was set. Charge state screening was enabled to exclude precursors with 0 and 1 charge states and 10 s dynamic exclusion was enabled (exclusion list size 500). For internal mass calibration the 445.120025 ion was used as lock mass. MS spectra were acquired with the Orbitrap in the range of m/z 400–1500 at a FWHM resolution of 15,000 at 400 m/z. The included m/z values were selected and HCD fragmentation was performed at 40 and 35% collision energies for two-charge and three charge states, respectively. The LTQ Orbitrap instrument was connected to an Ultimate 3000 RSLC (Dionex). Upon injection, the fraction was loaded onto the enrichment column (C18 PepMap100, 100 µm I.D, 100 Å pore size, 5 µm particle size) using 2% ACN, 0.1% FA. Then the analytical column (C18 PepMap100, 75 μm I.D, 100 Å pore size, 2 μm particle size) was switched in-line using a 35 min multi slope gradient as follows: 3.2% ACN at 4 min post injection, 20% at 8 min, 40% at 26 min and a 72% plateau before equilibration for 7 min.

#### Standard concentration curves

Targeted peptides for AP-F13A1 (tAP-F13A1), two light synthetic peptides and two heavy synthetic peptides containing L(^13^C_6_^15^N), R(^13^C_6_^15^N_4_), were obtained from Thermo Fisher Scientific with purity > 99% (Additional file 2). The two heavy peptides were as follows: AVPPNNSNAAEDDL*(^13^C_6_^15^N)PTVEL*(^13^C_6_^15^N)QGVVPR*(^13^C_6_^15^N_4_) (tAP-F13A1, valine variant) and AVPPNNSNAAEDDLPTVEL*(^13^C_6_^15^N)QGL*(^13^C_6_^15^N)VPR*(^13^C_6_^15^N_4_) (tAP-F13A1, leucine variant). Light and heavy peptides were spiked together to generate standard concentration curves keeping the heavy peptide at 52 ng/ml and spanning the light peptide from 13 to 208 ng/mL over six concentration points (13, 26, 52, 104, 169 and 208 ng/mL) Three replicates for each concentration points, from the lowest to the highest concentration, were acquired for both curves in the SPE simplified serum matrix. The matrix used for leucine and valine variant peptides were sera from homozygous patients V/V or L/L, respectively.

#### LC-PRM of tAP-F13A1 peptides

For LC-PRM assays, the instrument and the chromatographic method were the same as optimized previously in 2.4.2 except for some mass spectrometry settings. The LTQ-Velos Orbitrap was set up in data independent mode and using the following parameters for the detection of MS/MS spectra of tAP-F13A1 peptides (the HCD collision activation time of 10 ms, the isolation window of 3 m/z and a FWHM resolution of 7500 at 400 m/z during fragmentation) (Additional file 2a).

Alternatively, the Q-Exactive instrument was set-up as “targeted-MS2” method for the detection of tAP-F13A1 MS/MS spectra. The included m/z values were selected by the quadrupole with a maximum injection time of 100 ms and a maximum AGC target of 1e6. HCD fragmentation was performed at 25 and 29% collision energies for two and three charge states, respectively. Fragments were ejected from the HCD cell and read out in the Orbitrap mass analyzer at a FMWH resolution of 35,000 (Additional file 2b).

#### Data analysis

All quantifications of the tAP-F13A1 peptides were performed on Skyline (version 1.4) (http://proteome.gs.washington.edu/software/skyline) [12]. The peptide setting was set to trypsin as cleavage specificity, using no missed cleavages and 13C(6)15 N(4) C-term Arginine and 13C(6)15 N(1) Leucine as specific isotope modifications. In addition an MS/MS peptide spectra library generated from acquisition of tAP-F13A1 peptides on LTQ-Orbitrap and identified using Mascot (Matrix sciences) was included in the software (Additional file 3). The spectral library provides a quick and accurate procedure to match experimental MS/MS spectra with the collection available in the library yielding a dot product probability (dotp). Details on MS/MS spectra library are given in Additional file 3. The spectra filter options were set on two and three charge states for precursors, and the four most intense “y” fragments were selected for the MS/MS (y12, y11, y7 and y2; Fig. 2a). MS/MS spectra filtering was set up with an isolation width of m/z = 3 at a resolving power of 7500 (at 400 m/z) and Orbitrap as mass analyser. Both valine and leucine variants have been provided for review as part of user interface (Additional file 4). MS/MS spectra peak picking/integration and the identification/quantification of the precursors were done by matching with the spectral library. In addition, confident peak integration corresponding to the extracted ion chromatogram of the four most intense fragments of a selected precursor was adjusted manually to avoid potential interferences.

### Statistical analysis

Data plot columns, Wilcoxon test calculations and receiver operating characteristic (ROC) analysis between cancer and controls patients were performed using GraphPad Prism version 6.00 for Windows (GraphPad Software, San Diego, USA, www.graphpad.com).

## Results

### Peak candidate identification

LTQ Velos Orbitrap and Q-Exactive mass spectrometers were used to identify F13A1 peptides. Interestingly, identified peptides were found to represent the region of the activation peptide of the coagulation factor XIII A chain (F13A1), also known as the transglutaminase A chain (Additional file 5). MS/MS fragmentation highlighted the N-terminal acetylation (+ 42.011 Da) as the natural post-translational modification. It is of interest that this activation peptide of 37 amino acids possesses a natural Valine substitution by the Leucine (Val34Leu substitution) (Additional file 5a, b). At least, 8 tryptic-like fragments (proteoforms of the AP-FX13A1) were identified; (Acetyl)-SETSRTAFGGRRAVPPNNSNAAEDDLPTVELQGV(L)VPR (${\text{MH}}_{(Valine)}^{ + } =$ 3907.964 Da, ${\text{MH}}_{(Leucine)}^{ + } =$ 3921.980 Da and $MH_{(acetyl + Valine)}^{ + } = \, 3,949.011{\text{ Da}}$, $MH_{(acetyl + Leucine)}^{ + } = \, 3,963.991{\text{ Da}}$) TAFGGRRAVPPNNSNAAEDDLPTVELQGV(L)VPR (${\text{MH}}_{(Valine)}^{ + } =$ 3347.710 Da and ${\text{MH}}_{(Leucine)}^{ + } =$ 3361.723 Da), RAVPPNNSNAAEDDLPTVELQGV(L)VPR (${\text{MH}}_{(Valine)}^{ + } =$ 2758.408 Da and ${\text{MH}}_{(Leucine)}^{ + } =$ 2772.428) and AVPPNNSNAAEDDLPTVELQGV(L)VPR (${\text{MH}}_{(Valine)}^{ + } =$ 2602.314 Da and ${\text{MH}}_{(Leucine)}^{ + } =$ 2616.322) (Additional file 5c–h).

### Serum level of FXIII and FXIII-A by ELISA assays

**Fig. 1:**
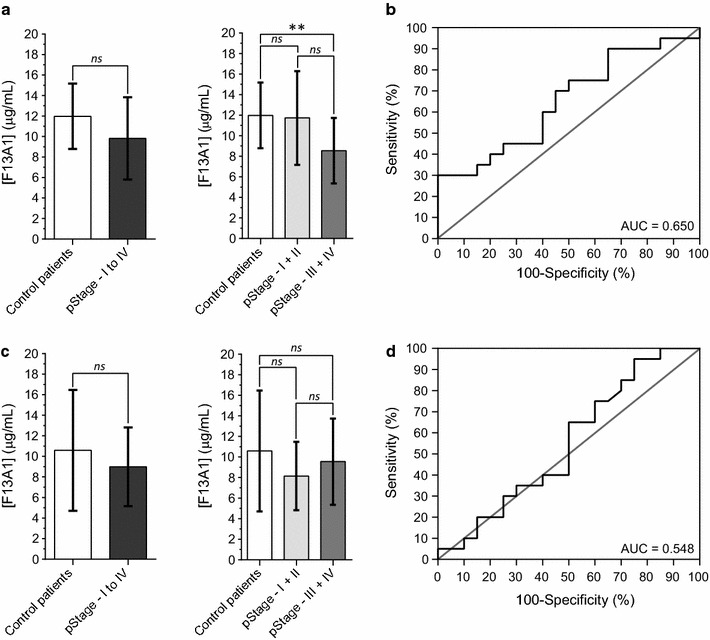
Serum concentration of FXIII in healthy patients and patients with declared CRC. Two different commercial ELISA were tested to determine the serum level of FXIII; **a, b** the Abcam ELISA assay (#ab108836) and **c**, **d** the ELISA from USCN (#E91094Hu). Receiver operating characteristic (ROC) curve of FXIII using the Abcam assay (**b**) and the USCN assay (**d**)

Serum levels of FXIII protein were estimated by two different ELISA assays in a cohort of 40 patients (see Table 1, Biobank 1). The Abcam ELISA assay (PID# ab108836) uses a polyclonal antibody for the detection of FXIII. The mean concentration detected in controls and CRC patients, were 11.98 ± 3.19 and 9.83 ± 4.01 μg/mL respectively, and were not significantly different by the Wilcoxon test calculation (*P* value = 0.06) (Fig. 1a). Only a significant regulation of FXIII was observed in serum of CRC patients when divided in two groups, according to the pStage I-II (*P* value = 0.46) and III-IV (**P value = 0.004) (Fig. 1a). However, the ROC curve area for the FXIII was 0.65 (95% CI = 0.82–0.48) discriminating patients with a low specificity (40%) and low sensitivity (60%) of detection at the cut-off value of 10.70 μg/mL (Fig. 1b). In the USCN ELISA assay (E91094Hu) another polyclonal antibody against the FXIII was used to determine specific serum level. The mean concentrations detected in controls and CRC patients, were 10.59 ± 5.88 and 9.99 ± 3.83 μg/mL respectively, were not significantly different by the Wilcoxon test calculation (*P* value = 0.26) (Fig. 1c). In addition, non-significant regulation of FXIII was observed in serum of CRC patients divided according to the pStage I-II and III-IV (Fig. 1c). The AUC calculated was 0.548 (95% CI = 0.73–0.36) by the use of the USCN ELISA assay. The calculated sensitivity and the specificity was 40 and 60% at the cut off value of 7.70 μg/mL (Fig. 1d).

### Quantitative PRM assay of AP-F13A1

#### Optimization of peptide detection by LC–MS

This qualitative step aims to optimize parameters for facilitating the development of an assay to allow absolute quantification using high-resolution mass spectrometry (LC PRM-Orbitrap). The isolation and enrichment of the AP-FX13A1 proteoforms were achieved by a C18 SPE cartridge. Only fragments and proteins with low molecular weights were bound to the hydrophobic phase. Trypsin digestion was carried out to combine all detected AP-F13A1 fragments identified in one smaller peptide facilitating the development of a quantification assay (Additional file 5a–h). Using this strategy, and according to the polymorphism of the AP-F13A1, only two targeted peptides were finally screened during LC-PRM analyses (AVPPNNSNAAEDDLPTVELQGLVPR and AVPPNNSNAAEDDLPTVELQGVVPR are tAP-F13A1 peptides). The chromatographic gradient was reduced to save valuable analysis time. The best compromise was an analysis cycle of 35 min with a useful gradient using a slope of 1.11% ACN/min from 20% ACN to 40% ACN in 18 min. Some settings were maximized on the high-resolution mass spectrometers. For the Q-Exactive, HCD fragmentation was used for the AVPPNNSNAAEDDLPTVELQGVVPR and the AVPPNNSNAAEDDLPTVELQGLVPR peptides. We further demonstrated that ionized peptides were available in two and three charge states. Depending on these observations, the HCD fragmentation has been maximized to 29% when the peptide counts a two-charge state and 25% for a three charge state. The inclusion list for LC-PRM experiments consisted of eight masses derived from the two targeted peptides (tAP-F13A1), the light and heavy forms; 868.108, 872.780, 876.122, 880.794, 1301.65, 1308.666, 1313.680, 1320.688 (Additional file 2a, b).

#### Skyline MS/MS filtering method

**Fig. 2:**
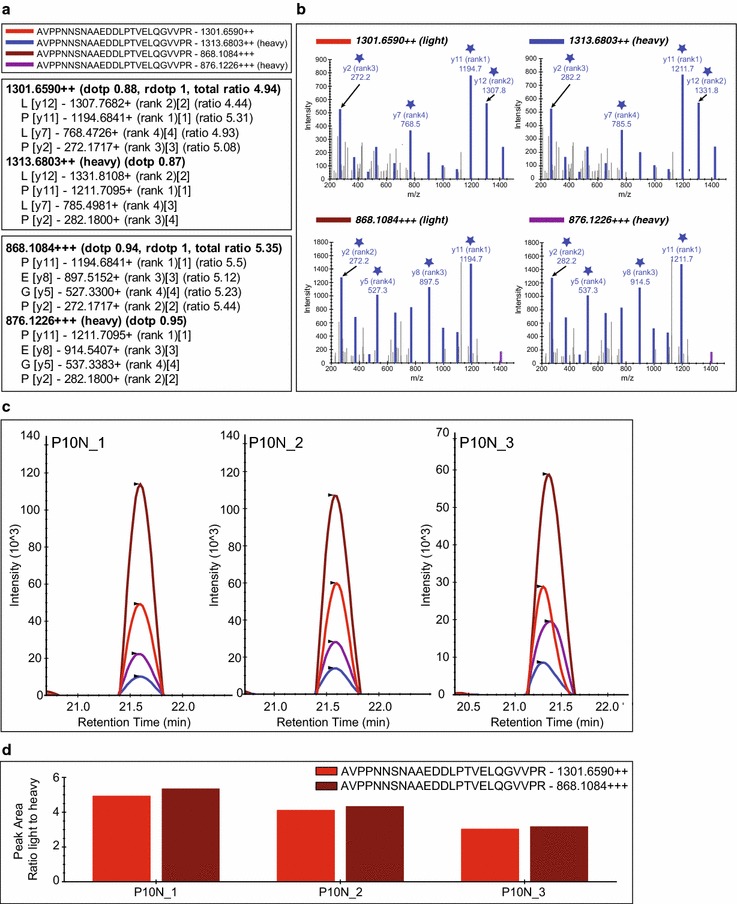
Skyline MS/MS filtering. **a** Skyline peptide tree for peptide AVPPNNSNAAEDDLPTVELQGVVPR with two (++) or three (+++) charges showing the four most intense extracted MS/MS fragments for light (1301.6590; 868.1084) and heavy precursors (1313.6803; 868.1084). **b** Skyline library of MS/MS spectra for the targeted peptides highlighting in blue matching fragments for an injected replicate. ★ highlight the four most intense extracted MS/MS fragments selected for the absolute quantification of AVPPNNSNAAEDDLPTVELQGVVPR **c** Chromatograms and peak intensity traces for three technical acquisitions of control patient (number accession P10) with heavy peptide spiked at 50 fmol/μL (130 ng/mL). **d** Peak area replicates normalized to the heavy peptide for the two (++) and three (+++) charged peptides (peak area light peptide/peak area heavy peptide)

The graphical interface for inspecting MS/MS filtered data using Skyline is shown in Fig. 2 illustrating the result for a homozygous patient Valine/Valine (control patient, P10). In this example, the dotp is between 0.87 and 1.00 showing an excellent matching of the experimental MS/MS request versus the MS/MS pattern available in the library (Fig. 2a, b and Additional files 3 and 4). tAP-F13A1 elutes around 21.5 min and the peak widths are less than 30 s, showing a perfect matching in term of retention time (Fig. 2c). The sum of the light/heavy ratios of the four “y” fragments intensities (y12, y11, y7 and y2), shows a high reproducibility between the 3 technical replicates (Fig. 2c, d). In addition, the coefficient of variation (CV) for the three technical acquisitions for the control patient is around 18% (accession number P10), suggesting a correct reproducibility of quantification (Additional file 6, panel C).

#### Standard concentration curves

**Fig. 3:**
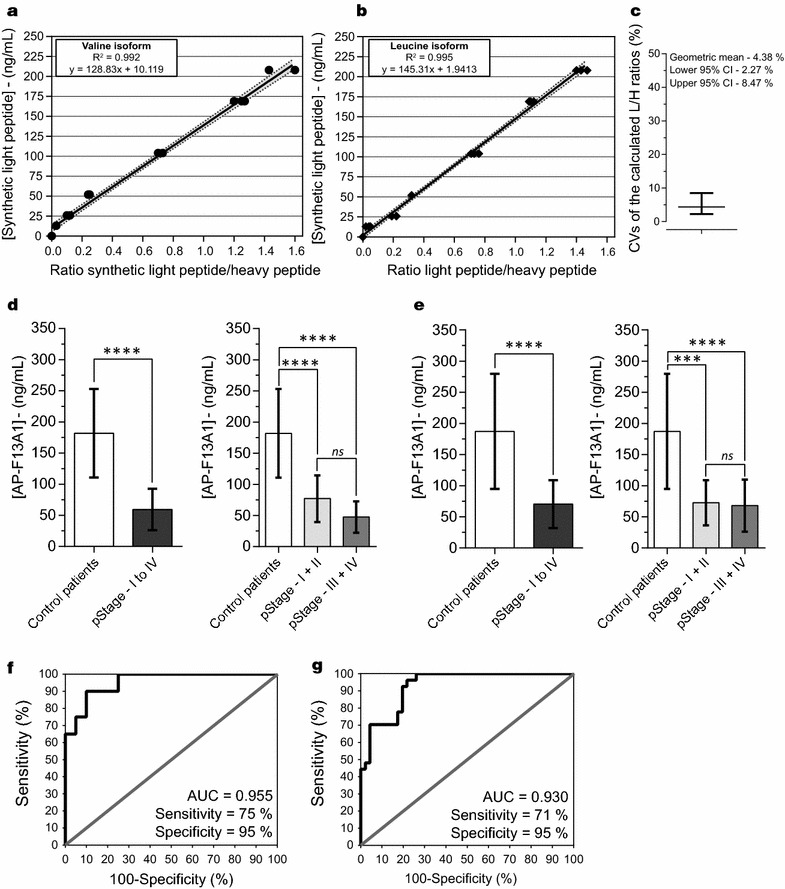
Calibration curves and evaluation of AP-F13A1 fragments by LC-PRM assays. **a, b** Graphical plot representation of theoretical concentration spanning according to the experimental peak ratio light/heavy peptides for AVPPNNSNAAEDDLPTVELQGVVPR and AVPPNNSNAAEDDLPTVELQGLVPR. **c** Coefficients of variations (CVs) of the light/heavy ratios distribution for the two peptides, over the 6 concentration points. Serum concentration of AP-F13A1 in healthy patients and patients with declared CRC from the first sample bank (**d**) and the second sample bank (**e**). The calculated means were 181.8 ± 71.0 and 187.2 ± 92.2 ng/mL for the controls, and 59.4 ± 33.3 and 70.4 ± 38.4 ng/mL for the cancer patients, both showing significant Wilcoxon tests (****P value = 0.0001). Receiver Operating Characteristic curves (ROC) for AP-F13A1 from first sample bank (**f**) and the second one (**g**). AUC computing were 0.95 (0.89–1.00) and 0.93 (0.87–0.98) and calculated values of sensitivity/specificity were 75%/90% and 71%/95%, respectively

To assess the reproducibility and robustness of the PRM assays and the bioinformatic Skyline processing, standard concentrations curves were generated in three replicates (Fig. 3a, b and Additional file 6, panel A). Light and heavy labeled tAP-F13A1 peptides were spiked in simplified serum to yield seven concentrations points ranging from 0 to 208 ng/mL. The simplified serum was processed from homozygous patients Valine/Valine or Leucine/Leucine to mimic the analytical matrix. These standard concentration curves demonstrate good linear regressions with good inter- and intra- analysis reproducibility. For each peptide monitored, the weighted regressions ranged from 0.992 (tAP-F13A1, valine variant) to 0.995 (tAP-F13A1, leucine variant). CVs of the light/heavy ratio peptide were under 10% except at the lowest concentration point (13 ng/mL) with CVs of 15 and 32% for the valine and the leucine variants, respectively (Additional file 6, Panel A). The geometric mean across all concentration points was 4.38%, suggesting an excellent reproducibility of analyses (Fig. 3c). For each tAP-F13A1, the lower limit of detection (LLOD) and the lower limit of quantification (LLOQ) were determined by the following statistical approach described by Keshishian and co-workers [13]. The LLOD and LLOQ values were determined at 6.3–18.9 and 7.4–22.2 ng/mL for AVPPNNSNNAAEDDlPTVELQGVVPR and AVPPNNSNNAAEDDlPTVELQGLVPR, respectively.

#### Quantification of AP-FX13A1 peptides by LC-PRM

To test the ability of the PRM assay to separate CRC patients from controls, we determined the concentration of AP-F13A1 variants in a first cohort of 40 volunteers (19 cancer patients and 21 controls) (Table 1, Biobank 1). Serum levels of tAP-F13A1 peptides, AVPPNNSNAAEDDLPTVELQGVVPR and AVPPNNSNAAEDDLPTVELQGLVPR, were determined by absolute quantification, using the AQUA approach. Absolute quantities were determined via the ratios of the endogenous peptides (light) and the heavy peptides provided by the Skyline MS/MS filtering processing. The standard concentration curve generated in the simplified serum was used for the quantification of tAP-F13A1. By considering the overall AP-F13A1 heterozygous patients (valine/leucine isoform) and homozygous patients (valine/valine or leucine/leucine isoforms), mean of AP-F13A1 were determined to be 181.8 ± 71.0 and 59.4 ± 33.3 ng/mL for control and CRC patients respectively which was significantly different (****P value < 0.0001) (Fig. 3d). Significant regulation of tAP-F13A1 was also observed in serum of CRC patients divided in two groups according to the pStage I-II and III-IV (Fig. 3d). For tAP-F13A1, the test showed a 95% specificity and 75% sensitivity when applying a cut-off of 95.7 ng/mL. Moreover the diagnostic accuracy of the test (AUC) for tAP-F13A1 was 0.955, suggesting that AP-FX13A1 peptides can be considered as potential biomarkers for the detection of CRC (Fig. 3f).

To confirm these initial results, we have determined the concentration of AP-F13A1 in a new cohort of 73 volunteers (27 cancer patients and 46 controls, Table 1; Biobank 2). Serum levels of AP-F13A1 were determined as previously described. Mean concentrations of AP-F13A1 were 187.2 ± 92.2 ng/mL (controls) and 70.4 ± 38.4 ng/mL (CRC) again showing significant differences between controls and CRC patients respectively (****P value ≤ 0.0001) (Fig. 3e). Significant regulation of tAP-F13A1 was again observed in serum of CRC patients when divided in two groups according to the pStage (Fig. 3e). For AP-F13A1, the test showed 95% specificity and 71% sensitivity when applying the same threshold as previously calculated. Moreover, the diagnostic accuracy of the test (AUC) for AP-F13A1 was 0.93 (Fig. 3g). The analyses of ROC curve confirm that AP-F13A1 peptides show promise as biomarkers for the detection of CRC. CVs from individual patients (3 technical injections) and overall concentrations calculated for each individual patient are available in Additional file 6, Panel B, C and Additional file 7.

## Discussion

Early screening for adenoma and CRC has decreased the incidence and mortality for CRC [14]. However, current methods such as iFOBT have poor sensitivity of detection in the asymptomatic population (27% sensitivity for adenoma, 65% sensitivity for carcinoma) [3]. Presently, there is no non-invasive alternative to FOBT testing in screening for CRC, and efforts are now directed toward the development of a diagnostic test, more sensitive, reliable and specific for CRC using blood markers. In our previous quantitative-based proteomics strategy, we identified a set of potential protein serum markers related to the progression of colorectal cancer [10]. Some of these potential markers (F9, F10, F12, F13A1, F13b, FGA, KLKB1, KNG1, PROC, SERPINA1, SERPINA5, SERPINC1, VWF) highlighted a de-regulation of the coagulation system. Known association between coagulation disorders and cancer dates back to 1865 and the Trousseau’s Syndrome, showing a number of coagulation abnormalities which increase tendency to both thrombosis and/or haemorrhage of patients with cancer [15–17]. Some protein markers of the coagulation system are often used as diagnostic and prognostic indicators of malignancy [18, 19]. Because F13A1 (FXIIIA) is one the last factors involved in the coagulation system that catalyse the polymeric Fibrin in cross-linked Fibrin complex [20], we needed to evaluate the F13A1 protein to get greater insight into coagulation abnormalities in patients with colorectal cancer.

First, FXIII was assessed by ELISA assays in the cohort of 40 patients (21 healthy patients and 19 patients with CRC; Table 1, Biobank 1). In both ELISA tests, the reduced expression of the FXIII were not significantly confirmed in the patients with CRC (Fig. 1). Then we demonstrated that it was possible to identify the activated peptide of FXIII (AP-F13A1) in SPE-depleted serum using high-resolution mass spectrometry and data dependent acquisition. At least 8 tryptic-like forms were identified from 25 to 37 amino acids (Additional file 5). We used these results to set up a targeted proteomic assay to quantify the F13A1 activation peptide (AP-F13A1) in the serum of patients. The development of a PRM assay of AP-F13A1 was possible by the optimization of sample preparation and mass spectrometry settings. The use of a solid phase extraction (SPE) facilitated the isolation and the enrichment of several low abundant proteins, such as cytokines/chemokines, peptides hormones along with proteolytic fragments of larger proteins, including the 8 proteoforms of AP-F13A1. The trypsin digestion allowed us to quantify of all these proteoforms by the selection of the smallest tryptic peptides AVPPNNSNAAEDDLPTVELQGV(L)VPR. The quantification settings were validated in several experimental processes, including the step of accurate inclusion mass screening [21]. Calibration curves were determined using synthetic light and heavy peptides, spiked into the simplified serum matrix and using the Q-Exactive mass spectrometer. For the two peptides, the range was obtained over 2 orders of magnitude (Q-Exactive linear slope of calibration curves equal to 0.991 and 0.995) (Fig. 3a, b). The performance of the method was first evaluated in the same cohort of 40 volunteers and then confirmed by a cohort of 73 patients from a second biobank (Table 1). The quantification of the two isoforms of AP-F13A1 demonstrated significant discrimination between controls and patients with CRC, indicating that AP-F13A1 is down-regulated during progression of CRC (Fig. 3).

The present study supports the concept that ELISA can be limiting in the validation process of candidate biomarkers coming from proteomic analysis. ELISAs used in our study were designed to target either the tetrameric proenzyme FXIII, F13A1 and F13B but not the specific AP-F13A1. To date, there are no suitable antibodies available for specific screening of the released forms of AP-F13A1 in the serum of patients through ELISA assays. In addition, ELISAs require development for each form of AP-F13A1 (Leucine or Valine isoforms), which both are time consuming and expensive. Other recent studies have demonstrated the value of targeted proteomics in screening for cancer biomarkers during the preclinical evaluation and clinical validation [6, 9, 22, 23]. A possible important advantage over immunoassays was the ability to include and compare the two different AP-F13A1 isoforms in a single PRM experiment. Our data have shown that PRM-based evaluation of the different forms of AP-F13A1 from patients with CRC can be used in the clinical environment. In addition, further proteotypic peptides can be added to the existing PRM assay, in order to gradually assemble a panel of candidate biomarkers that are relevant to the clinical question.

The coagulation factor XIII (FXIII) is a tetrameric proenzyme (FXIII-A_2_B_2_) retrieved in the blood, and consists of two A subunits (F13A1) and two B subunits (F13B) with different molecular functions after the release of AP-F13A1. The mean concentration of the proenzyme was reported to be 14–28 μg/mL [24]. Our ELISA studies confirmed similar levels of FXIII expression in the blood of patients (11.98 ± 3.19 and 10.59 ± 5.88 μg/mL). F13A1 also known as transglutaminase chain A, has enzymatic activity and belongs to the transglutaminase family. According to the Uniprot entry (http://www.uniprot.org/uniprot/P00488), F13A1 consists of 731 amino acids (83 kDa) which is acetylated (+ 42.010 Da) on the N-terminal of the mature molecule. The major source of F13A1 comes from bone marrow cells, but it has also been detected in other cell types such as megakaryocytes, thrombocytes, monocytes, alveolar macrophages and tumor-associated macrophages [25]. During activation of F13A1, thrombin cleaves the activation peptide from the N-terminus by hydrolyzing the Arg37-Gly38 peptide bond. Other proteases such as MASP1, F10, or trypsin have been shown to cleave the Arg37-Gly38 peptide bond at lower rate [26]. It is interesting to note that ELISA assays revealed average concentration of FXIII protein around 10 µg/ml three log higher than the AP-F13A1 concentration revealed by our PRM assays. The decrease of serum AP-F13A1 in CRC patients could be explained by the inhibition of these proteases that cleave the activation peptide of the coagulation F13A1 or the result of consumption of AP-F13A1 by proteases of the hemostasis process within the bed of the ulcerated cancer. Supporting this hypothesis, we found some tryptic-like forms of AP-F13A1 in patients’ serum. Alternatively, some matrix effects in our biological groups could explain this decrease, for example carrier proteins in serum could bind the AP-F13A1 in CRC patients and then be eliminated by our SPE initial steps. However, the use of acid condition for the SPE step is more likely to break peptide/protein interactions.

F13A1 is the key regulator of coagulation machinery, strengthens the fibrin clot by cross-linking fibrin chains and protecting fibrin chains from the fibrinolysis [20]. In addition, F13A1 exerts other physiological effects associated with wound-healing, tissue remodeling and tissue turnover [27]. It has been demonstrated that F13A1 is involved in stimulation of endothelial cell proliferation, migration, inhibition of apoptosis and exhibits a proangiogenic activity associated with the inhibition of THSB1 [28]. Although such properties may promote tumorigenesis, little is presently known about F13A1 and the consequences of AP-F13A1 polymorphism in human cancer. It has been reported, in the Caucasian population, ~ 38% are heterozygous for the two isoforms, ~ 56% are homozygous for the valine isoform, and ~ 6% are homozygous for the leucine isoform [29, 30]. In the 113 patients included in our study, a similar distribution was determined: 28% are heterozygous for the two forms, 62% are homozygous for the valine isoform and 10% are homozygous for the leucine isoform. The prevalence of AP-F13A1 polymorphism has been investigated and influence the rate of the FXIII cleavage showing an impact on fibrin clot [31], but was probably not associated with venous thrombosis and progression of adenomatous polyps and CRC [32]. However, in a large population-based CRC study, Vossen et al. [29] have recently found a 15% risk reduction for heterozygous patients of AP-F13A1. We still cannot confirm the hypothesis of Vossen et al., because of the small number of samples included in our study (113 patients). However, the decrease in AP-F13A1 observed between controls and patients with CRC in our study would be an indicator of the development and progression of CRC toward a stage with a less favorable prognosis.

## Conclusion

In summary, we have detected and quantified for the first time AP-F13A1 in patients with CRC and presented the proof of principle that in vivo release of AP-F13A1 can be measured by PRM-based assay. While acknowledging the limitations of these two small cohorts of patients, we conclude that the measurement of serum AP-F13A1 shows potential in distinguishing healthy patients from patients with CRC. Further studies are required to elucidate the underlying molecular mechanisms in coagulation-dependent signalling pathways in colorectal cancer biology.

## Additional files

**Additional file 1.** Workflow of the study from the biomarker discovery step to the validation of tAP-F13A1 by LC-PRM.

**Additional file 2.** Table of synthetic light and heavy stables isotope for tAP-F13A1 during inclusion mass screening and PRM-based assays.

**Additional file 3.** MS/MS Spectra from the Mascot DAT file imported in Skyline software as MS/MS library.

**Additional file 4.** MS/MS Filtering–transition setting tabs in Skyline software.

**Additional file 5.** Representative mass spectra of the AP-F13A1 activation peptide.

**Additional file 6.** Coefficients of Variation (CVs) of the ratios light/heavy.

**Additional file 7.** Table summary of patients selected for the absolute quantification of the two isoforms of tAP-F13A1 by LC-PRM.
