# Supplementary material for: Activation peptide of the coagulation factor XIII (AP-F13A1) as a new biomarker for the screening of colorectal cancer
Source: Clin Proteomics. 2018 Apr 9;15:15. doi: 10.1186/s12014-018-9191-3 (PMC5890357; doi:10.1186/s12014-018-9191-3)

Additional file 1
Workflow of the study from the biomarker discovery step to the validation of tAP-F13A1 by LC-PRM

F13A1 peaks detected during the discovery step were confirmed during high resolution mass spectrometry analysis (HRMS, 1). In parallel, serum is depleted of abundant and high molecular weight proteins by C18 solid phase extraction (SPE) to collect fragments available in the serum of patients (2). The panel of candidate biomarkers for AP-F13A1 obtained during the attribution of differential peaks were used for the verification and optimatisation of HRMS settings (3) to generate a list of targets LC-PRM assays (m/z, z, 4). Note that some peptides may be represented by more than one target on the list if multiple charge states are expected.

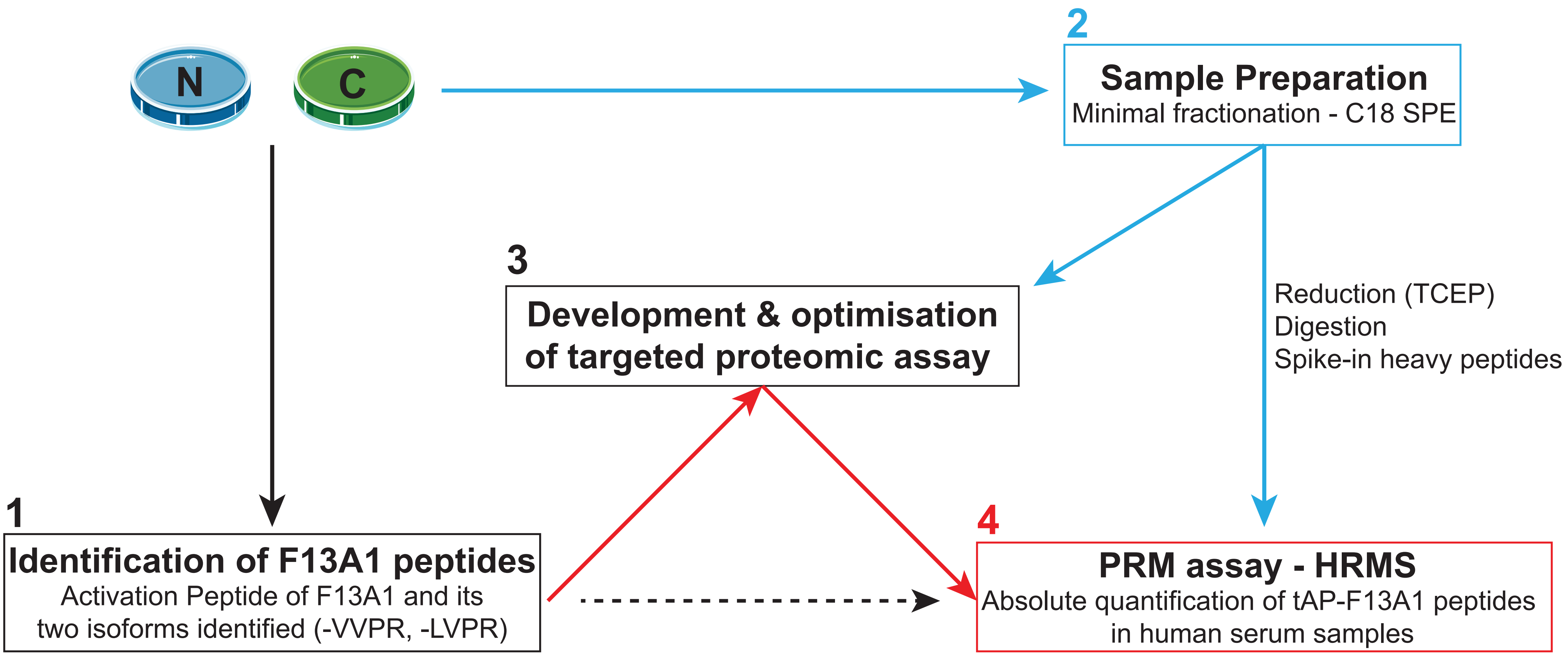

Supplement: Supplementary file 1 — Additional file 1. Workflow of the study from the biomarker discovery step to the validation of tAP-F13A1 by LC-PRM. [file 12014_2018_9191_MOESM1_ESM.pdf]
