# Supplementary material for: Activation peptide of the coagulation factor XIII (AP-F13A1) as a new biomarker for the screening of colorectal cancer
Source: Clin Proteomics. 2018 Apr 9;15:15. doi: 10.1186/s12014-018-9191-3 (PMC5890357; doi:10.1186/s12014-018-9191-3)
Supplement: Supplementary file 2 — Additional file 2. Table of synthetic light and heavy stables isotope for tAP-F13A1 during inclusion mass screening and PRM-based assays. [file 12014_2018_9191_MOESM2_ESM.pdf]

Additional file 2
Table of synthetic light and heavy stables isotope for tAP-F13A1 during inclussion mass screening and PRM-based assays

a - LTQ Orbitrap settings maximized for a better detection of tAP-F13A1

b - Q-Exactive settings maximized for a better detection of tAP-F13A1

a

tAP-F13A1 isoforms	Classification	Precursor [M]	Type of fragmentation	Collision energy	MS resolution	MS/MS resolution	Isolation width (m/z)
AVPPNNSNAAEDDLPTVELQGVVPR	Light	1301.659 ++	HCD	40	15,000	7,500	3
		868.1084 +++	HCD	35	15,000	7,500	3
AVPPNNSNAAEDD**L**($^{13}C_6{}^{15}N$)PTVE**L**($^{13}C_6{}^{15}N$)QGVVP**R**($^{13}C_6{}^{15}N_4$)	Heavy	1313.6803 ++	HCD	40	15,000	7,500	3
		876.1226 +++	HCD	35	15,000	7,500	3
AVPPNNSNAAEDDLPTVELQGLVPR	Light	1308.6668 ++	HCD	40	15,000	7,500	3
		872.7803 +++	HCD	35	15,000	7,500	3
AVPPNNSNAAEDDLPTVE**L**($^{13}C_6{}^{15}N$)QG**L**($^{13}C_6{}^{15}N$)VP**R**($^{13}C_6{}^{15}N_4$)	Heavy	1320.6881 ++	HCD	40	15,000	7,500	3
		880.7945 +++	HCD	35	15,000	7,500	3

b

tAP-F13A1 isoforms	Classification	Precursor [M]	Type of fragmentation	Collision energy	MS/MS resolution	Isolation width (m/z)
AVPPNNSNAAEDDLPTVELQGVVPR	Light	1301.659 ++	HCD	29	35,000	3
		868.1084 +++	HCD	25	35,000	3
AVPPNNSNAAEDD**L**($^{13}C_6{}^{15}N$)PTVE**L**($^{13}C_6{}^{15}N$)QGVVP**R**($^{13}C_6{}^{15}N_4$)	Heavy	1313.6803 ++	HCD	29	35,000	3
		876.1226 +++	HCD	25	35,000	3
AVPPNNSNAAEDDLPTVELQGLVPR	Light	1308.6668 ++	HCD	29	35,000	3
		872.7803 +++	HCD	25	35,000	3
AVPPNNSNAAEDDLPTVE**L**($^{13}C_6{}^{15}N$)QG**L**($^{13}C_6{}^{15}N$)VP**R**($^{13}C_6{}^{15}N_4$)	Heavy	1320.6881 ++	HCD	29	35,000	3
		880.7945 +++	HCD	25	35,000	3
