# Supplementary material for: Activation peptide of the coagulation factor XIII (AP-F13A1) as a new biomarker for the screening of colorectal cancer
Source: Clin Proteomics. 2018 Apr 9;15:15. doi: 10.1186/s12014-018-9191-3 (PMC5890357; doi:10.1186/s12014-018-9191-3)
Supplement: Supplementary file 3 — Additional file 3. MS/MS Spectra from the Mascot DAT file imported in Skyline software as MS/MS library. [file 12014_2018_9191_MOESM3_ESM.pdf]

# Additional file 3
# MS/MS Spectra from the Mascot DAT file imported in Skyline software as MS/MS library

MS/MS spectra of the two isoforms of tAP-F13A1 obtained from serum samples using the C18 solid phase extraction.

**a** - MS/MS spectrum of tAP-F13A1 AVPPNNSNAAEDDLPTVELQGVVPR with the precursor ion M at m/z 1301.6590 ++

**b** - MS/MS spectrum of tAP-F13A1 AVPPNNSNAAEDDLPTVELQGVVPR with the precursor ion M at m/z 868.1084 +++

**c** - MS/MS spectrum of tAP-F13A1 AVPPNNSNAAEDDLPTVELQGLVPR with the precursor ion M at m/z 1308.6680 ++

**d** - MS/MS spectrum of tAP-F13A1 AVPPNNSNAAEDDLPTVELQGLVPR with the precursor ion M at m/z 872.7803 +++

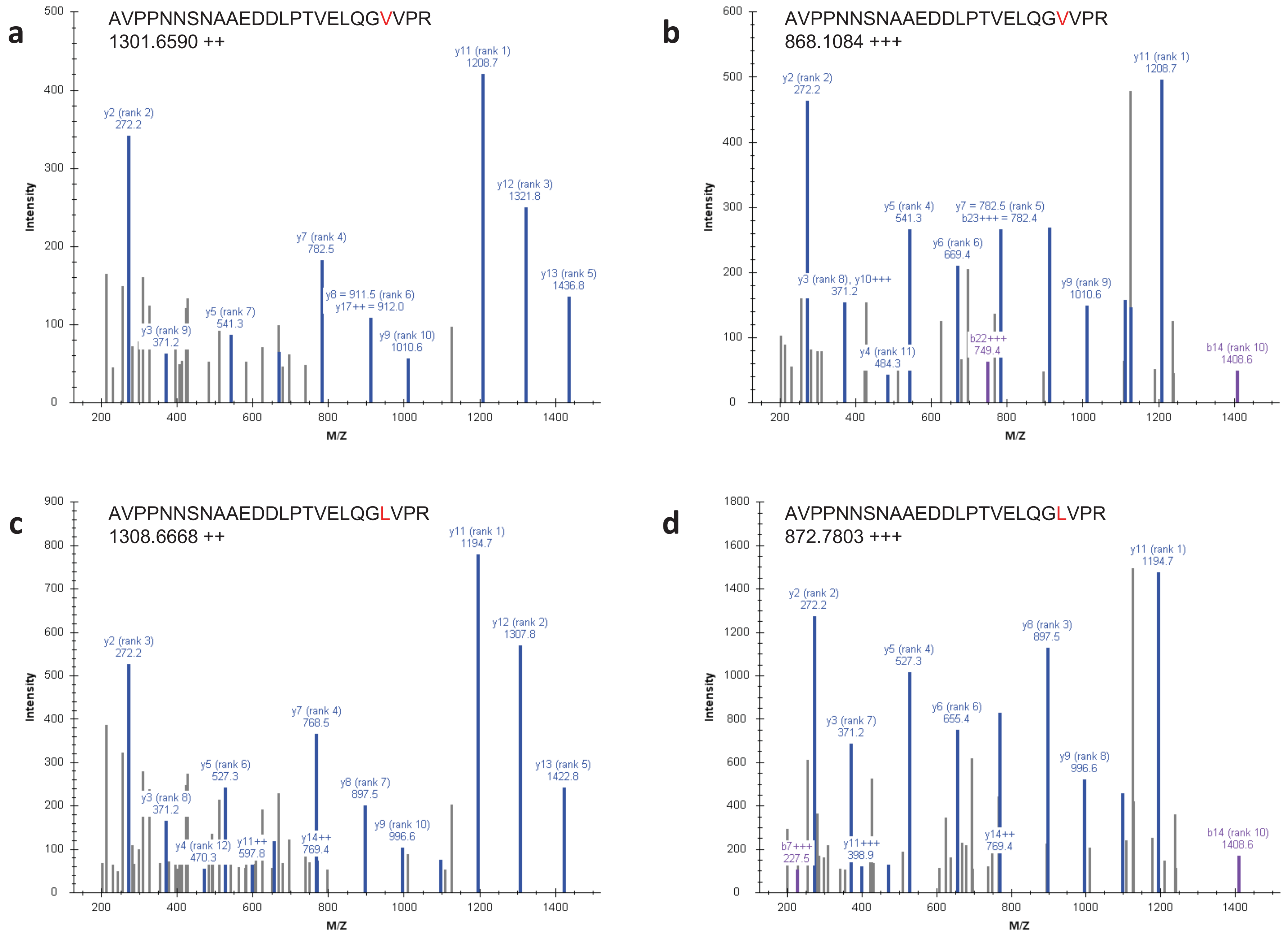

a
AVPPNNSNAAEDDLPTVELQGVVPR
1301.6590 ++
y2 (rank 2) 272.2
y3 (rank 9) 371.2
y5 (rank 7) 541.3
y7 (rank 4) 782.5
y8 = 911.5 (rank 6) y17++ = 912.0
y9 (rank 10) 1010.6
y11 (rank 1) 1208.7
y12 (rank 3) 1321.8
y13 (rank 5) 1436.8
Intensity
M/Z
b
AVPPNNSNAAEDDLPTVELQGVVPR
868.1084 +++
y2 (rank 2) 272.2
y3 (rank 8), y10+++ 371.2
y4 (rank 11) 484.3
y5 (rank 4) 541.3
y6 (rank 6) 669.4
b22+++ 749.4
y7 = 782.5 (rank 5) b23+++ = 782.4
y9 (rank 9) 1010.6
y11 (rank 1) 1208.7
b14 (rank 10) 1408.6
Intensity
M/Z
c
AVPPNNSNAAEDDLPTVELQGLVPR
1308.6668 ++
y2 (rank 3) 272.2
y3 (rank 8) 371.2
y4 (rank 12) 470.3
y5 (rank 6) 527.3
y11++ 597.8
y7 (rank 4) 768.5
y14++ 769.4
y8 (rank 7) 897.5
y9 (rank 10) 996.6
y11 (rank 1) 1194.7
y12 (rank 2) 1307.8
y13 (rank 5) 1422.8
Intensity
M/Z
d
AVPPNNSNAAEDDLPTVELQGLVPR
872.7803 +++
b7+++ 227.5
y2 (rank 2) 272.2
y3 (rank 7) 371.2
y11+++ 398.9
y5 (rank 4) 527.3
y6 (rank 6) 655.4
y14++ 769.4
y8 (rank 3) 897.5
y9 (rank 8) 996.6
y11 (rank 1) 1194.7
b14 (rank 10) 1408.6
Intensity
M/Z
