# Supplementary material for: Activation peptide of the coagulation factor XIII (AP-F13A1) as a new biomarker for the screening of colorectal cancer
Source: Clin Proteomics. 2018 Apr 9;15:15. doi: 10.1186/s12014-018-9191-3 (PMC5890357; doi:10.1186/s12014-018-9191-3)
Supplement: Supplementary file 4 — Additional file 4. MS/MS Filtering–transition setting tabs in Skyline software. [file 12014_2018_9191_MOESM4_ESM.pdf]

Additional file 4
MS/MS Filtering - transition setting tabs in Skyline software

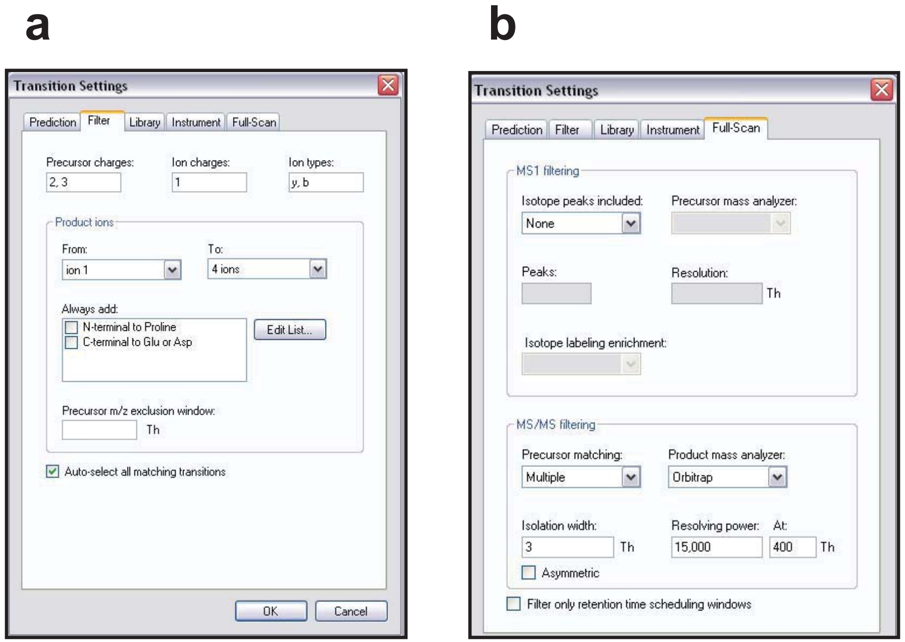

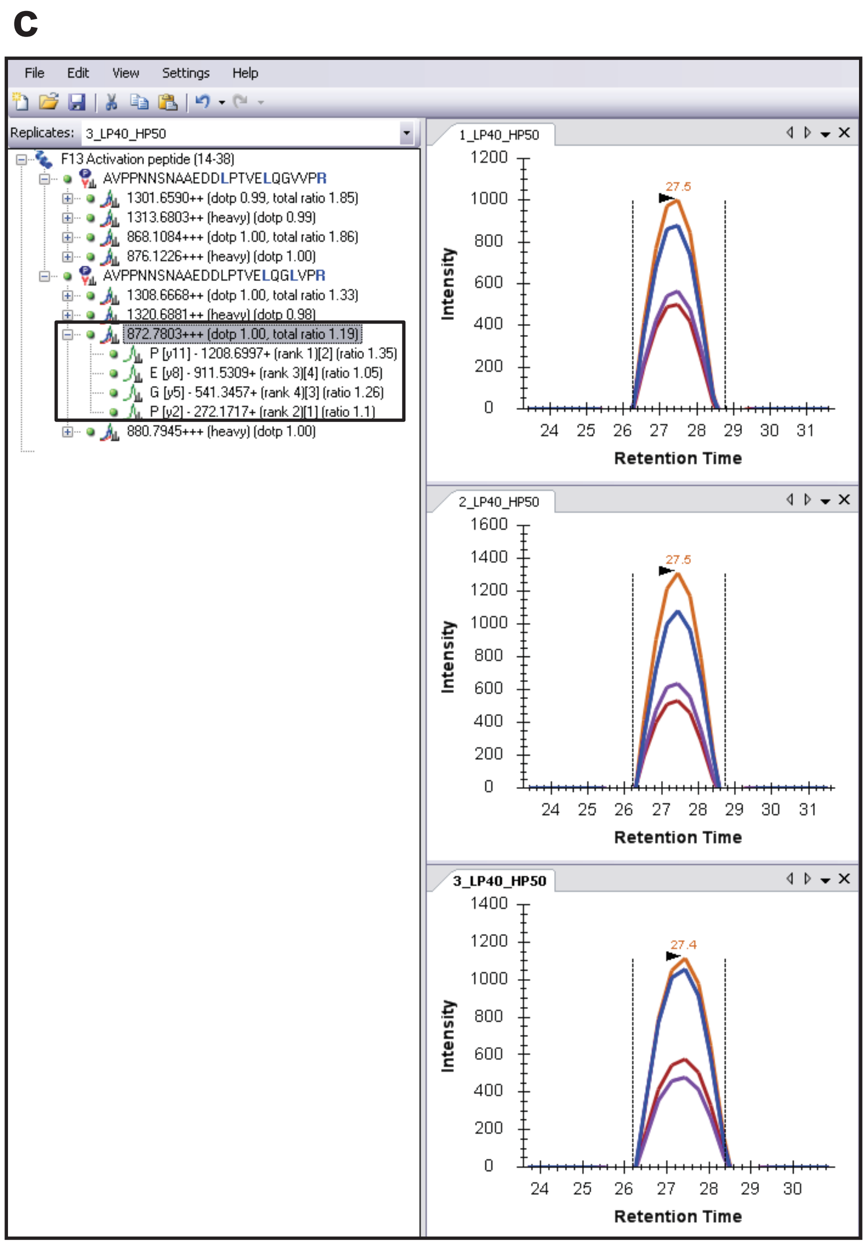

a. Skyline MS2 filtering Options and Settings used in the study. Filter features are given by the selected charge states of the precusor (++, +++), the type of fragment product y ion and the number of fragment product ions you want to screen (the first most intense ion to the fourth).

b. Skyline Full-Scan option, the MS/MS settings that control chromatogram extraction from MS/MS scans of multiple precusor matching read out by the orbitrap with an isolation width of 3 m/z and a fixed resolution of 15,000 at 400 m/z.

c. Peptide AVPPNNSNAAEDDLPTVELQGLVPR +++ and the fourth intense extracted fragment product y-ions at m/z 1208.6997, 911.5309, 541.3457 and 272.1717 are displayed for three triplicate injections
