# Supplementary material for: Activation peptide of the coagulation factor XIII (AP-F13A1) as a new biomarker for the screening of colorectal cancer
Source: Clin Proteomics. 2018 Apr 9;15:15. doi: 10.1186/s12014-018-9191-3 (PMC5890357; doi:10.1186/s12014-018-9191-3)
Supplement: Supplementary file 5 — Additional file 5. Representative mass spectra of the AP-F13A1 activation peptide. [file 12014_2018_9191_MOESM5_ESM.pdf]

Additional file 5
Representative mass spectra of the AP-F13A1 activation peptide

a - Annotated MS/MS spectrum of the Acetyl-SETSRTAFGGRRAVPPNNSNAAEDDLPTVELQGVVPR peptide (MH^+ = 3,949.981, m/z = 988.251 (z=4)), identified as the first largest form of the activation peptide of the coagulation factor XIII (AP-F13A1)

b - Annotated MS/MS spectrum of the Acetyl-SETSRTAFGGRRAVPPNNSNAAEDDLPTVELQGLVPR peptide (MH^+ = 3,963.9938, m/z = 991.753 (z=4)), identified as the second largest form of the AP-F13A1

c - Annotated MS/MS spectrum of the TAFGGRRAVPPNNSNAAEDDLPTVELQGVVPR peptide (MH^+ = 3,347.710, m/z = 837.683 (z=4)), identified as one of the intermediate form of the AP-F13A1

d - Annotated MS/MS spectrum of the TAFGGRRAVPPNNSNAAEDDLPTVELQGL VPR peptide (MH^+ = 3,361.723, m/z = 841.186 (z=4)), identified as one of the intermediate form of the AP-F13A1

e - Annotated MS/MS spectrum of the RAVPPNNSNAAEDDLPTVELQGVVPR peptide (MH^+ = 2,758.408, m/z = 920.141 (z=3)), identified as one of the intermediate form of the AP-F13A1

f - Annotated MS/MS spectrum of the RAVPPNNSNAAEDDLPTVELQGLVPR peptide (MH^+ = 2,772.428, m/z = 924.814 (z=3)), identified as one of the intermediate form of the AP-F13A1

g - Annotated MS/MS spectrum of the AVPPNNSNAAEDDLPTVELQGVVPR peptide (MH^+ = 2,602.314, m/z = 868.109 (z=3)), identified as the shortest form of the AP-F13A1

h - Annotated MS/MS spectrum of the AVPPNNSNAAEDDLPTVELQGLVPR peptide (MH^+ = 2,616.322, m/z = 872.779 (z=3)), identified as the shortest form of the AP-F13A1

a

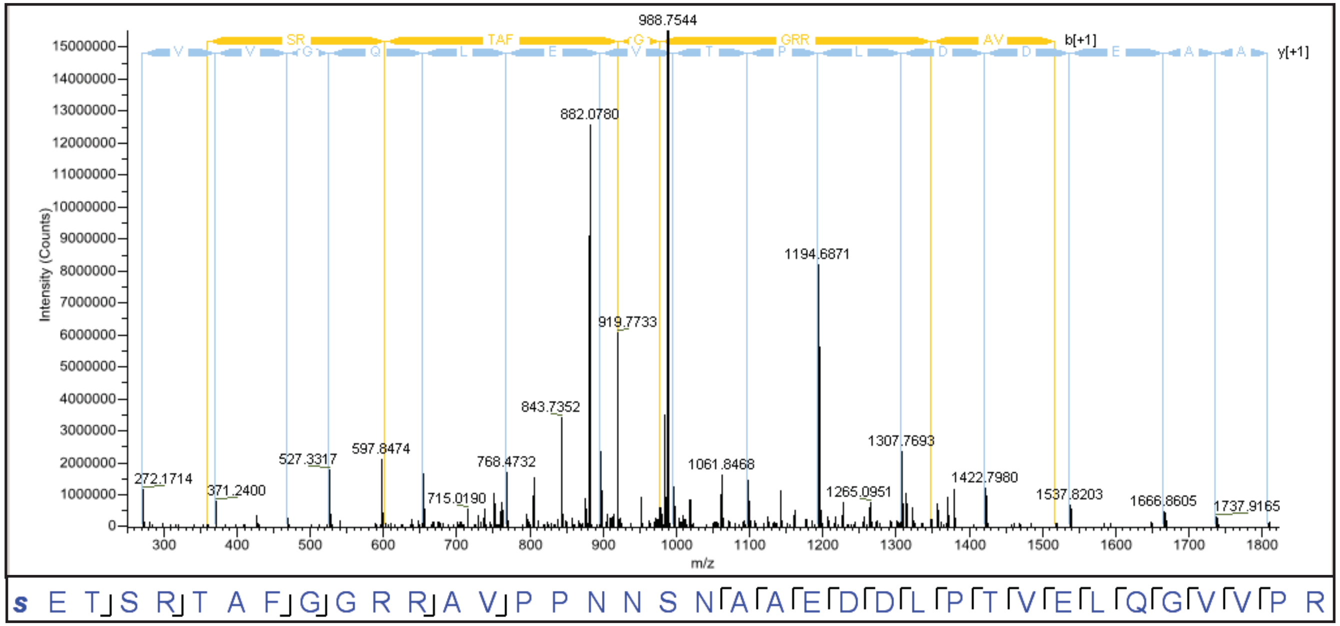

#1	b⁺	b²⁺	b³⁺	b⁴⁺	Seq.	y⁺	y²⁺	y³⁺	y⁴⁺	#2
1	130.04987	65.52857	44.02147	33.26793	S-Acetyl					37
2	259.09247	130.04987	87.03567	65.52858	E	3820.93270	1910.96999	1274.31575	955.98863	36
3	360.14015	180.57371	120.71823	90.79050	T	3691.89010	1846.44869	1231.30155	923.72798	35
4	447.17218	224.08973	149.72891	112.54850	S	3590.84242	1795.92485	1197.61899	898.46606	34
5	603.27330	302.14029	201.76262	151.57378	R	3503.81039	1752.40883	1168.60831	876.70806	33
6	704.32098	352.66413	235.44518	176.83570	T	3347.70927	1674.35827	1116.57461	837.68278	32
7	775.35810	388.18269	259.12422	194.59498	A	3246.66159	1623.83443	1082.89205	812.42086	31
8	922.42652	461.71690	308.14702	231.36209	F	3175.62447	1588.31587	1059.21301	794.66158	30
9	979.44799	490.22763	327.15418	245.61746	G	3028.55605	1514.78166	1010.19020	757.89447	29
10	1036.46946	518.73837	346.16134	259.87282	G	2971.53458	1486.27093	991.18304	743.63910	28
11	1192.57058	596.78893	398.19504	298.89810	R	2914.51311	1457.76019	972.17589	729.38374	27
12	1348.67170	674.83949	450.22875	337.92338	R	2758.41199	1379.70963	920.14218	690.35846	26
13	1419.70882	710.35805	473.90779	355.68266	A	2602.31087	1301.65907	868.10847	651.33318	25
14	1518.77724	759.89226	506.93060	380.44977	V	2531.27375	1266.14051	844.42943	633.57390	24
15	1615.83001	808.41864	539.28152	404.71296	P	2432.20533	1216.60630	811.40663	608.80679	23
16	1712.88278	856.94503	571.63244	428.97615	P	2335.15256	1168.07992	779.05570	584.54360	22
17	1826.92571	913.96649	609.64675	457.48689	N	2238.09979	1119.55353	746.70478	560.28041	21
18	1940.96864	970.98796	647.66106	485.99762	N	2124.05686	1062.53207	708.69047	531.76967	20
19	2028.00067	1014.50397	676.67174	507.75563	S	2010.01393	1005.51060	670.67616	503.25894	19
20	2142.04360	1071.52544	714.68605	536.26636	N	1922.98190	961.99459	641.66548	481.50093	18
21	2213.08072	1107.04400	738.36509	554.02564	A	1808.93897	904.97312	603.65117	452.99020	17
22	2284.11784	1142.56256	762.04413	571.78492	A	1737.90185	869.45456	579.97213	435.23092	16
23	2413.16044	1207.08386	805.05833	604.04557	E	1666.86473	833.93600	556.29309	417.47164	15
24	2528.18739	1264.59733	843.40065	632.80231	D	1537.82213	769.41470	513.27889	385.21099	14
25	2643.21434	1322.11081	881.74296	661.55904	D	1422.79518	711.90123	474.93658	356.45425	13
26	2756.29841	1378.65284	919.43765	689.83006	L	1307.76823	654.38775	436.59426	327.69752	12
27	2853.35118	1427.17923	951.78858	714.09325	P	1194.68416	597.84572	398.89957	299.42650	11
28	2954.39886	1477.70307	985.47114	739.35517	T	1097.63139	549.31933	366.54865	275.16331	10
29	3053.46728	1527.23728	1018.49394	764.12228	V	996.58371	498.79549	332.86609	249.90139	9
30	3182.50988	1591.75858	1061.50814	796.38293	E	897.51529	449.26128	299.84328	225.13428	8
31	3295.59395	1648.30061	1099.20283	824.65395	L	768.47269	384.73998	256.82908	192.87363	7
32	3423.65253	1712.32990	1141.88903	856.66859	Q	655.38862	328.19795	219.13439	164.60261	6
33	3480.67400	1740.84064	1160.89618	870.92396	G	527.33004	264.16866	176.44820	132.58797	5
34	3579.74242	1790.37485	1193.91899	895.69106	V	470.30857	235.65792	157.44104	118.33260	4
35	3678.81084	1839.90906	1226.94180	920.45817	V	371.24015	186.12371	124.41823	93.56550	3
36	3775.86361	1888.43544	1259.29272	944.72136	P	272.17173	136.58950	91.39543	68.79839	2
37					R	175.11896	88.06312	59.04450	44.53520	1

b

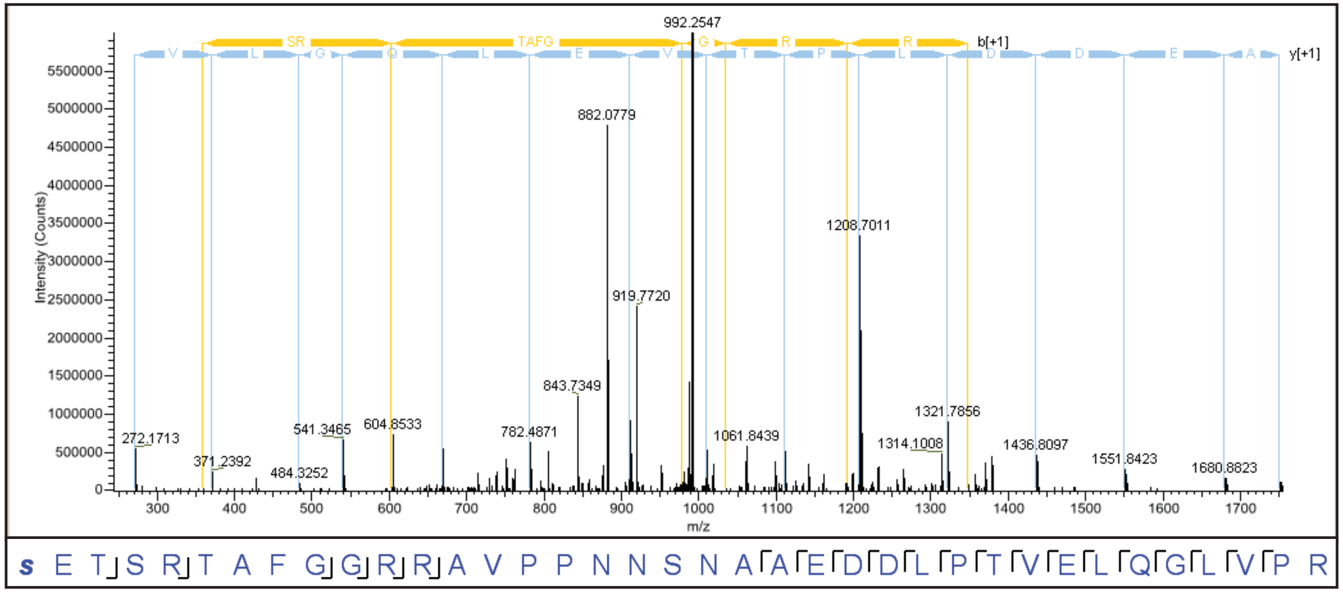

#1	b⁺	b²⁺	b³⁺	b⁴⁺	Seq.	y⁺	y²⁺	y³⁺	y⁴⁺	#2
1	130.04987	65.52857	44.02147	33.26793	S-Acetyl					37
2	259.09247	130.04987	87.03567	65.52858	E	3834.94835	1917.97781	1278.98763	959.49255	36
3	360.14015	180.57371	120.71823	90.79050	T	3705.90575	1853.45651	1235.97343	927.23190	35
4	447.17218	224.08973	149.72891	112.54850	S	3604.85807	1802.93267	1202.29087	901.96998	34
5	603.27330	302.14029	201.76262	151.57378	R	3517.82604	1759.41666	1173.28020	880.21197	33
6	704.32098	352.66413	235.44518	176.83570	T	3361.72492	1681.36610	1121.24649	841.18669	32
7	775.35810	388.18269	259.12422	194.59498	A	3260.67724	1630.84226	1087.56393	815.92477	31
8	922.42652	461.71690	308.14702	231.36209	F	3189.64012	1595.32370	1063.88489	798.16549	30
9	979.44799	490.22763	327.15418	245.61746	G	3042.57170	1521.78949	1014.86208	761.39838	29
10	1036.46946	518.73837	346.16134	259.87282	G	2985.55023	1493.27875	995.85493	747.14302	28
11	1192.57058	596.78893	398.19504	298.89810	R	2928.52876	1464.76802	976.84777	732.88765	27
12	1348.67170	674.83949	450.22875	337.92338	R	2772.42764	1386.71746	924.81406	693.86237	26
13	1419.70882	710.35805	473.90779	355.68266	A	2616.32652	1308.66690	872.78036	654.83709	25
14	1518.77724	759.89226	506.93060	380.44977	V	2545.28940	1273.14834	849.10132	637.07781	24
15	1615.83001	808.41864	539.28152	404.71296	P	2446.22098	1223.61413	816.07851	612.31070	23
16	1712.88278	856.94503	571.63244	428.97615	P	2349.16821	1175.08774	783.72759	588.04751	22
17	1826.92571	913.96649	609.64675	457.48689	N	2252.11544	1126.56136	751.37666	563.78432	21
18	1940.96864	970.98796	647.66106	485.99762	N	2138.07251	1069.53989	713.36235	535.27359	20
19	2028.00067	1014.50397	676.67174	507.75563	S	2024.02958	1012.51843	675.34804	506.76285	19
20	2142.04360	1071.52544	714.68605	536.26636	N	1936.99755	969.00241	646.33737	485.00485	18
21	2213.08072	1107.04400	738.36509	554.02564	A	1822.95462	911.98095	608.32306	456.49411	17
22	2284.11784	1142.56256	762.04413	571.78492	A	1751.91750	876.46239	584.64402	438.73483	16
23	2413.16044	1207.08386	805.05833	604.04557	E	1680.88038	840.94383	560.96498	420.97555	15
24	2528.18739	1264.59733	843.40065	632.80231	D	1551.83778	776.42253	517.95078	388.71490	14
25	2643.21434	1322.11081	881.74296	661.55904	D	1436.81083	718.90905	479.60846	359.95817	13
26	2756.29841	1378.65284	919.43765	689.83006	L	1321.78388	661.39558	441.26614	331.20143	12
27	2853.35118	1427.17923	951.78858	714.09325	P	1208.69981	604.85354	403.57145	302.93041	11
28	2954.39886	1477.70307	985.47114	739.35517	T	1111.64704	556.32716	371.22053	278.66722	10
29	3053.46728	1527.23728	1018.49394	764.12228	V	1010.59936	505.80332	337.53797	253.40530	9
30	3182.50988	1591.75858	1061.50814	796.38293	E	911.53094	456.26911	304.51516	228.63819	8
31	3295.59395	1648.30061	1099.20283	824.65395	L	782.48834	391.74781	261.50096	196.37754	7
32	3423.65253	1712.32990	1141.88903	856.66859	Q	669.40427	335.20577	223.80627	168.10653	6
33	3480.67400	1740.84064	1160.89618	870.92396	G	541.34569	271.17648	181.12008	136.09188	5
34	3593.75807	1797.38267	1198.59087	899.19498	L	484.32422	242.66575	162.11292	121.83651	4
35	3692.82649	1846.91688	1231.61368	923.96208	V	371.24015	186.12371	124.41823	93.56550	3
36	3789.87926	1895.44327	1263.96460	948.22527	P	272.17173	136.58950	91.39543	68.79839	2
37					R	175.11896	88.06312	59.04450	44.53520	1

c

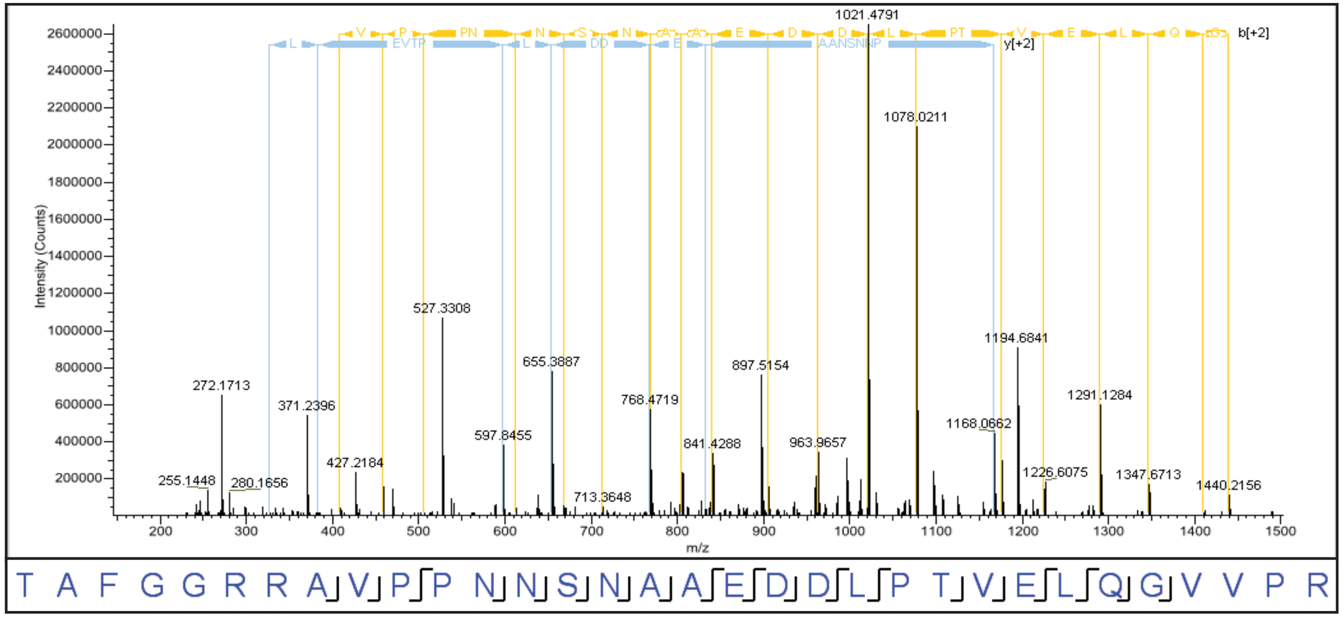

#1	b⁺	b²⁺	b³⁺	b⁴⁺	Seq.	y⁺	y²⁺	y³⁺	y⁴⁺	#2
1	102.05496	51.53112	34.68984	26.26920	T					32
2	173.09208	87.04968	58.36888	44.02848	A	3246.66159	1623.83443	1082.89205	812.42086	31
3	320.16050	160.58389	107.39168	80.79558	F	3175.62447	1588.31587	1059.21301	794.66158	30
4	377.18197	189.09462	126.39884	95.05095	G	3028.55605	1514.78166	1010.19020	757.89447	29
5	434.20344	217.60536	145.40600	109.30632	G	2971.53458	1486.27093	991.18304	743.63910	28
6	590.30456	295.65592	197.43970	148.33160	R	2914.51311	1457.76019	972.17589	729.38374	27
7	746.40568	373.70648	249.47341	187.35688	R	2758.41199	1379.70963	920.14218	690.35846	26
8	817.44280	409.22504	273.15245	205.11616	A	2602.31087	1301.65907	868.10847	651.33318	25
9	916.51122	458.75925	306.17526	229.88326	V	2531.27375	1266.14051	844.42943	633.57390	24
10	1013.56399	507.28563	338.52618	254.14645	P	2432.20533	1216.60630	811.40663	608.80679	23
11	1110.61676	555.81202	370.87710	278.40965	P	2335.15256	1168.07992	779.05570	584.54360	22
12	1224.65969	612.83348	408.89141	306.92038	N	2238.09979	1119.55353	746.70478	560.28041	21
13	1338.70262	669.85495	446.90572	335.43111	N	2124.05686	1062.53207	708.69047	531.76967	20
14	1425.73465	713.37096	475.91640	357.18912	S	2010.01393	1005.51060	670.67616	503.25894	19
15	1539.77758	770.39243	513.93071	385.69985	N	1922.98190	961.99459	641.66548	481.50093	18
16	1610.81470	805.91099	537.60975	403.45913	A	1808.93897	904.97312	603.65117	452.99020	17
17	1681.85182	841.42955	561.28879	421.21841	A	1737.90185	869.45456	579.97213	435.23092	16
18	1810.89442	905.95085	604.30299	453.47906	E	1666.86473	833.93600	556.29309	417.47164	15
19	1925.92137	963.46432	642.64531	482.23580	D	1537.82213	769.41470	513.27889	385.21099	14
20	2040.94832	1020.97780	680.98762	510.99254	D	1422.79518	711.90123	474.93658	356.45425	13
21	2154.03239	1077.51983	718.68231	539.26355	L	1307.76823	654.38775	436.59426	327.69752	12
22	2251.08516	1126.04622	751.03324	563.52675	P	1194.68416	597.84572	398.89957	299.42650	11
23	2352.13284	1176.57006	784.71580	588.78867	T	1097.63139	549.31933	366.54865	275.16331	10
24	2451.20126	1226.10427	817.73860	613.55577	V	996.58371	498.79549	332.86609	249.90139	9
25	2580.24386	1290.62557	860.75280	645.81642	E	897.51529	449.26128	299.84328	225.13428	8
26	2693.32793	1347.16760	898.44749	674.08744	L	768.47269	384.73998	256.82908	192.87363	7
27	2821.38651	1411.19689	941.13369	706.10208	Q	655.38862	328.19795	219.13439	164.60261	6
28	2878.40798	1439.70763	960.14084	720.35745	G	527.33004	264.16866	176.44820	132.58797	5
29	2977.47640	1489.24184	993.16365	745.12456	V	470.30857	235.65792	157.44104	118.33260	4
30	3076.54482	1538.77605	1026.18646	769.89166	V	371.24015	186.12371	124.41823	93.56550	3
31	3173.59759	1587.30243	1058.53738	794.15485	P	272.17173	136.58950	91.39543	68.79839	2
32					R	175.11896	88.06312	59.04450	44.53520	1

d

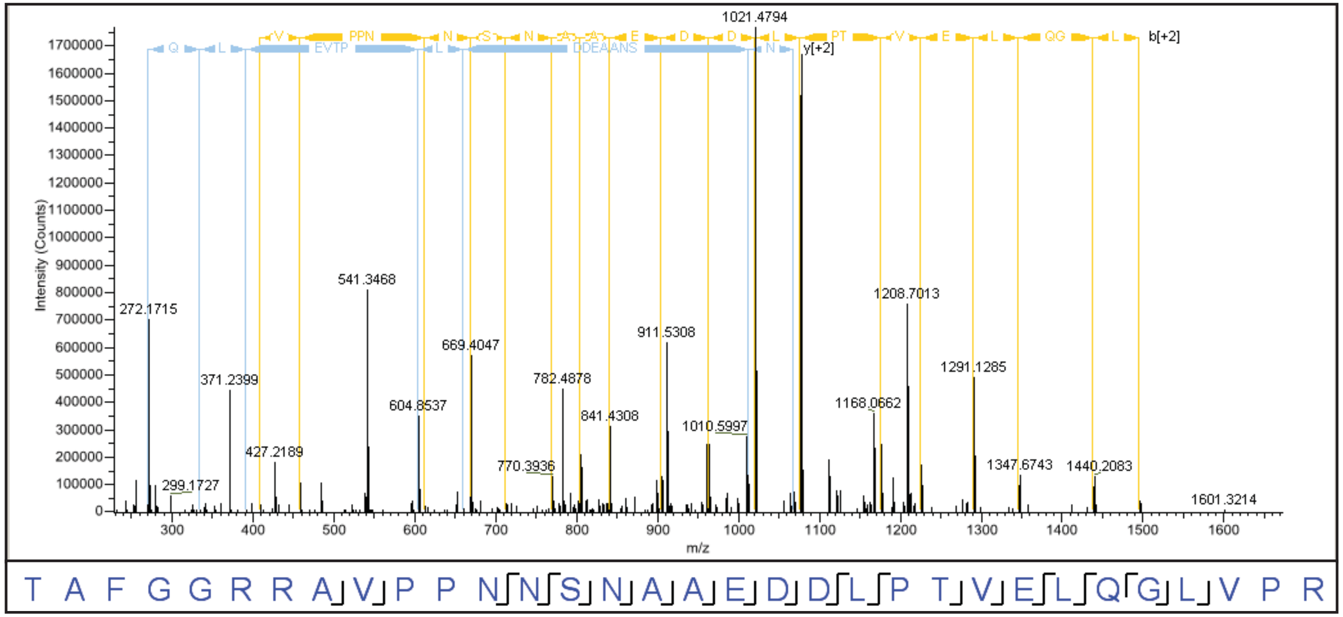

#1	b⁺	b²⁺	b³⁺	b⁴⁺	Seq.	y⁺	y²⁺	y³⁺	y⁴⁺	#2
1	102.05496	51.53112	34.68984	26.26920	T					32
2	173.09208	87.04968	58.36888	44.02848	A	3260.67724	1630.84226	1087.56393	815.92477	31
3	320.16050	160.58389	107.39168	80.79558	F	3189.64012	1595.32370	1063.88489	798.16549	30
4	377.18197	189.09462	126.39884	95.05095	G	3042.57170	1521.78949	1014.86208	761.39838	29
5	434.20344	217.60536	145.40600	109.30632	G	2985.55023	1493.27875	995.85493	747.14302	28
6	590.30456	295.65592	197.43970	148.33160	R	2928.52876	1464.76802	976.84777	732.88765	27
7	746.40568	373.70648	249.47341	187.35688	R	2772.42764	1386.71746	924.81406	693.86237	26
8	817.44280	409.22504	273.15245	205.11616	A	2616.32652	1308.66690	872.78036	654.83709	25
9	916.51122	458.75925	306.17526	229.88326	V	2545.28940	1273.14834	849.10132	637.07781	24
10	1013.56399	507.28563	338.52618	254.14645	P	2446.22098	1223.61413	816.07851	612.31070	23
11	1110.61676	555.81202	370.87710	278.40965	P	2349.16821	1175.08774	783.72759	588.04751	22
12	1224.65969	612.83348	408.89141	306.92038	N	2252.11544	1126.56136	751.37666	563.78432	21
13	1338.70262	669.85495	446.90572	335.43111	N	2138.07251	1069.53989	713.36235	535.27359	20
14	1425.73465	713.37096	475.91640	357.18912	S	2024.02958	1012.51843	675.34804	506.76285	19
15	1539.77758	770.39243	513.93071	385.69985	N	1936.99755	969.00241	646.33737	485.00485	18
16	1610.81470	805.91099	537.60975	403.45913	A	1822.95462	911.98095	608.32306	456.49411	17
17	1681.85182	841.42955	561.28879	421.21841	A	1751.91750	876.46239	584.64402	438.73483	16
18	1810.89442	905.95085	604.30299	453.47906	E	1680.88038	840.94383	560.96498	420.97555	15
19	1925.92137	963.46432	642.64531	482.23580	D	1551.83778	776.42253	517.95078	388.71490	14
20	2040.94832	1020.97780	680.98762	510.99254	D	1436.81083	718.90905	479.60846	359.95817	13
21	2154.03239	1077.51983	718.68231	539.26355	L	1321.78388	661.39558	441.26614	331.20143	12
22	2251.08516	1126.04622	751.03324	563.52675	P	1208.69981	604.85354	403.57145	302.93041	11
23	2352.13284	1176.57006	784.71580	588.78867	T	1111.64704	556.32716	371.22053	278.66722	10
24	2451.20126	1226.10427	817.73860	613.55577	V	1010.59936	505.80332	337.53797	253.40530	9
25	2580.24386	1290.62557	860.75280	645.81642	E	911.53094	456.26911	304.51516	228.63819	8
26	2693.32793	1347.16760	898.44749	674.08744	L	782.48834	391.74781	261.50096	196.37754	7
27	2821.38651	1411.19689	941.13369	706.10208	Q	669.40427	335.20577	223.80627	168.10653	6
28	2878.40798	1439.70763	960.14084	720.35745	G	541.34569	271.17648	181.12008	136.09188	5
29	2991.49205	1496.24966	997.83553	748.62847	L	484.32422	242.66575	162.11292	121.83651	4
30	3090.56047	1545.78387	1030.85834	773.39557	V	371.24015	186.12371	124.41823	93.56550	3
31	3187.61324	1594.31026	1063.20926	797.65877	P	272.17173	136.58950	91.39543	68.79839	2
32					R	175.11896	88.06312	59.04450	44.53520	1

e

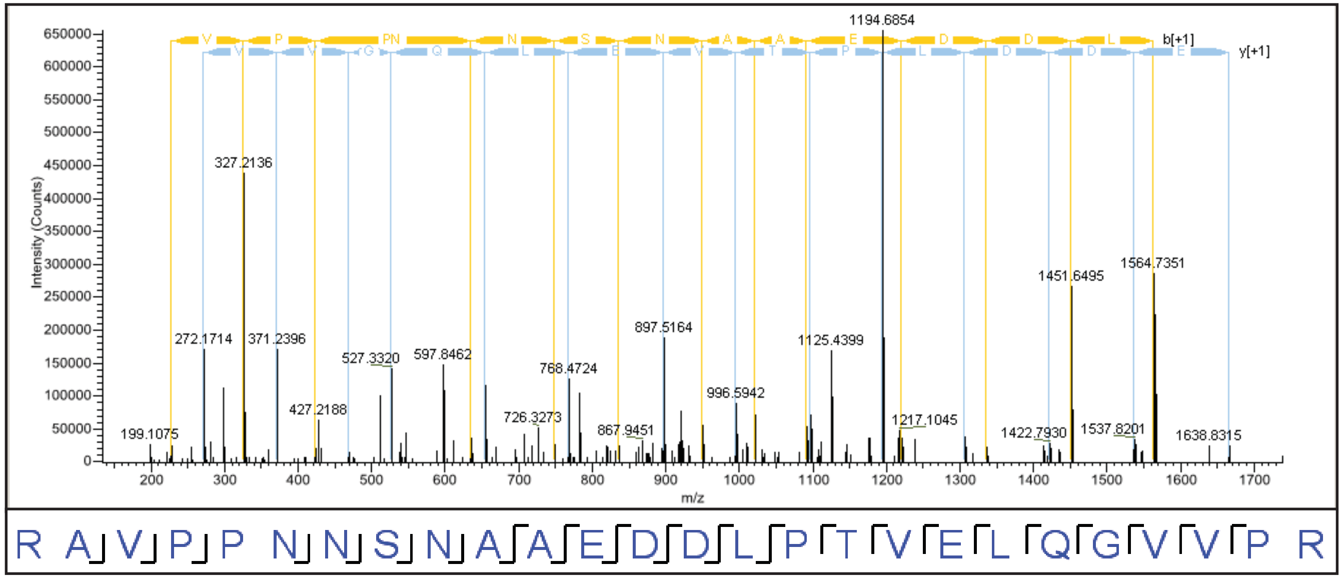

#1	b⁺	b²⁺	b³⁺	Seq.	y⁺	y²⁺	y³⁺	#2
1	157.10840	79.05784	53.04098	R				26
2	228.14552	114.57640	76.72002	A	2602.31087	1301.65907	868.10847	25
3	327.21394	164.11061	109.74283	V	2531.27375	1266.14051	844.42943	24
4	424.26671	212.63699	142.09375	P	2432.20533	1216.60630	811.40663	23
5	521.31948	261.16338	174.44468	P	2335.15256	1168.07992	779.05570	22
6	635.36241	318.18484	212.45899	N	2238.09979	1119.55353	746.70478	21
7	749.40534	375.20631	250.47330	N	2124.05686	1062.53207	708.69047	20
8	836.43737	418.72232	279.48397	S	2010.01393	1005.51060	670.67616	19
9	950.48030	475.74379	317.49828	N	1922.98190	961.99459	641.66548	18
10	1021.51742	511.26235	341.17732	A	1808.93897	904.97312	603.65117	17
11	1092.55454	546.78091	364.85636	A	1737.90185	869.45456	579.97213	16
12	1221.59714	611.30221	407.87056	E	1666.86473	833.93600	556.29309	15
13	1336.62409	668.81568	446.21288	D	1537.82213	769.41470	513.27889	14
14	1451.65104	726.32916	484.55520	D	1422.79518	711.90123	474.93658	13
15	1564.73511	782.87119	522.24989	L	1307.76823	654.38775	436.59426	12
16	1661.78788	831.39758	554.60081	P	1194.68416	597.84572	398.89957	11
17	1762.83556	881.92142	588.28337	T	1097.63139	549.31933	366.54865	10
18	1861.90398	931.45563	621.30618	V	996.58371	498.79549	332.86609	9
19	1990.94658	995.97693	664.32038	E	897.51529	449.26128	299.84328	8
20	2104.03065	1052.51896	702.01507	L	768.47269	384.73998	256.82908	7
21	2232.08923	1116.54825	744.70126	Q	655.38862	328.19795	219.13439	6
22	2289.11070	1145.05899	763.70842	G	527.33004	264.16866	176.44820	5
23	2388.17912	1194.59320	796.73122	V	470.30857	235.65792	157.44104	4
24	2487.24754	1244.12741	829.75403	V	371.24015	186.12371	124.41823	3
25	2584.30031	1292.65379	862.10495	P	272.17173	136.58950	91.39543	2
26				R	175.11896	88.06312	59.04450	1

f

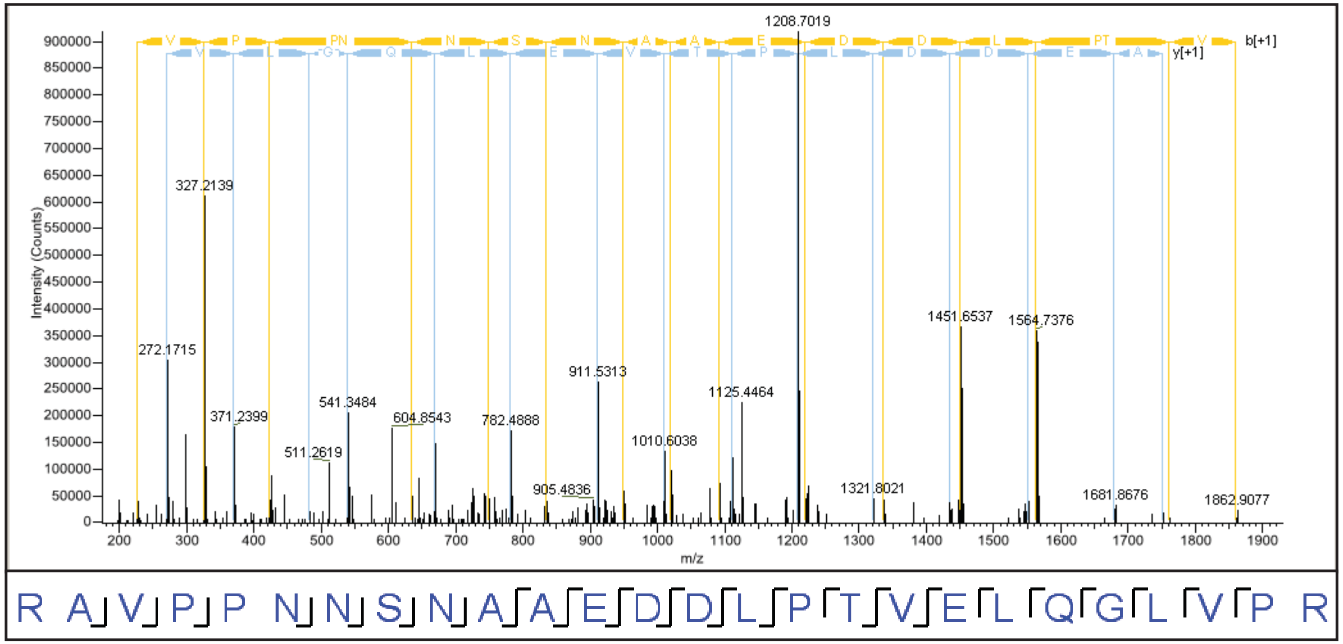

#1	b⁺	b²⁺	b³⁺	Seq.	y⁺	y²⁺	y³⁺	#2
1	157.10840	79.05784	53.04098	R				26
2	228.14552	114.57640	76.72002	A	2616.32652	1308.66690	872.78036	25
3	327.21394	164.11061	109.74283	V	2545.28940	1273.14834	849.10132	24
4	424.26671	212.63699	142.09375	P	2446.22098	1223.61413	816.07851	23
5	521.31948	261.16338	174.44468	P	2349.16821	1175.08774	783.72759	22
6	635.36241	318.18484	212.45899	N	2252.11544	1126.56136	751.37666	21
7	749.40534	375.20631	250.47330	N	2138.07251	1069.53989	713.36235	20
8	836.43737	418.72232	279.48397	S	2024.02958	1012.51843	675.34804	19
9	950.48030	475.74379	317.49828	N	1936.99755	969.00241	646.33737	18
10	1021.51742	511.26235	341.17732	A	1822.95462	911.98095	608.32306	17
11	1092.55454	546.78091	364.85636	A	1751.91750	876.46239	584.64402	16
12	1221.59714	611.30221	407.87056	E	1680.88038	840.94383	560.96498	15
13	1336.62409	668.81568	446.21288	D	1551.83778	776.42253	517.95078	14
14	1451.65104	726.32916	484.55520	D	1436.81083	718.90905	479.60846	13
15	1564.73511	782.87119	522.24989	L	1321.78388	661.39558	441.26614	12
16	1661.78788	831.39758	554.60081	P	1208.69981	604.85354	403.57145	11
17	1762.83556	881.92142	588.28337	T	1111.64704	556.32716	371.22053	10
18	1861.90398	931.45563	621.30618	V	1010.59936	505.80332	337.53797	9
19	1990.94658	995.97693	664.32038	E	911.53094	456.26911	304.51516	8
20	2104.03065	1052.51896	702.01507	L	782.48834	391.74781	261.50096	7
21	2232.08923	1116.54825	744.70126	Q	669.40427	335.20577	223.80627	6
22	2289.11070	1145.05899	763.70842	G	541.34569	271.17648	181.12008	5
23	2402.19477	1201.60102	801.40311	L	484.32422	242.66575	162.11292	4
24	2501.26319	1251.13523	834.42591	V	371.24015	186.12371	124.41823	3
25	2598.31596	1299.66162	866.77684	P	272.17173	136.58950	91.39543	2
26				R	175.11896	88.06312	59.04450	1

g

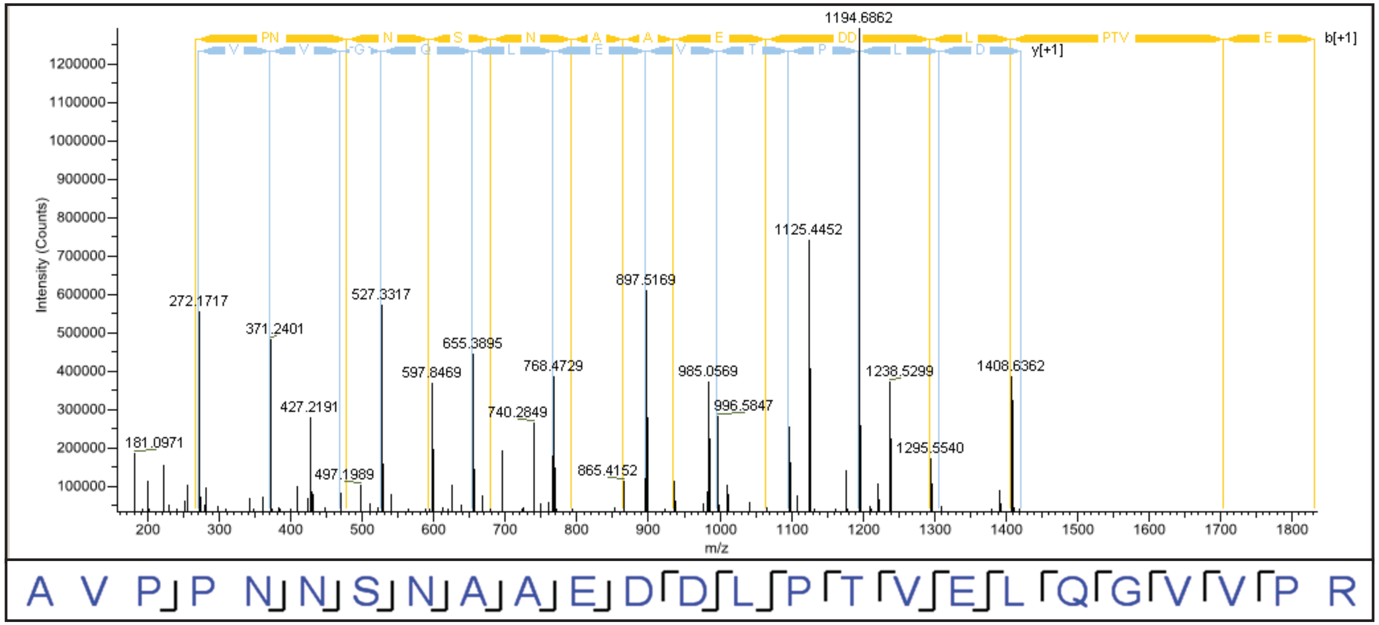

#1	b⁺	b²⁺	b³⁺	Seq.	y⁺	y²⁺	y³⁺	#2
1	72.04440	36.52584	24.68632	A				25
2	171.11282	86.06005	57.70912	V	2531.27375	1266.14051	844.42943	24
3	268.16559	134.58643	90.06005	P	2432.20533	1216.60630	811.40663	23
4	365.21836	183.11282	122.41097	P	2335.15256	1168.07992	779.05570	22
5	479.26129	240.13428	160.42528	N	2238.09979	1119.55353	746.70478	21
6	593.30422	297.15575	198.43959	N	2124.05686	1062.53207	708.69047	20
7	680.33625	340.67176	227.45027	S	2010.01393	1005.51060	670.67616	19
8	794.37918	397.69323	265.46458	N	1922.98190	961.99459	641.66548	18
9	865.41630	433.21179	289.14362	A	1808.93897	904.97312	603.65117	17
10	936.45342	468.73035	312.82266	A	1737.90185	869.45456	579.97213	16
11	1065.49602	533.25165	355.83686	E	1666.86473	833.93600	556.29309	15
12	1180.52297	590.76512	394.17917	D	1537.82213	769.41470	513.27889	14
13	1295.54992	648.27860	432.52149	D	1422.79518	711.90123	474.93658	13
14	1408.63399	704.82063	470.21618	L	1307.76823	654.38775	436.59426	12
15	1505.68676	753.34702	502.56710	P	1194.68416	597.84572	398.89957	11
16	1606.73444	803.87086	536.24966	T	1097.63139	549.31933	366.54865	10
17	1705.80286	853.40507	569.27247	V	996.58371	498.79549	332.86609	9
18	1834.84546	917.92637	612.28667	E	897.51529	449.26128	299.84328	8
19	1947.92953	974.46840	649.98136	L	768.47269	384.73998	256.82908	7
20	2075.98811	1038.49769	692.66755	Q	655.38862	328.19795	219.13439	6
21	2133.00958	1067.00843	711.67471	G	527.33004	264.16866	176.44820	5
22	2232.07800	1116.54264	744.69752	V	470.30857	235.65792	157.44104	4
23	2331.14642	1166.07685	777.72032	V	371.24015	186.12371	124.41823	3
24	2428.19919	1214.60323	810.07125	P	272.17173	136.58950	91.39543	2
25				R	175.11896	88.06312	59.04450	1

h

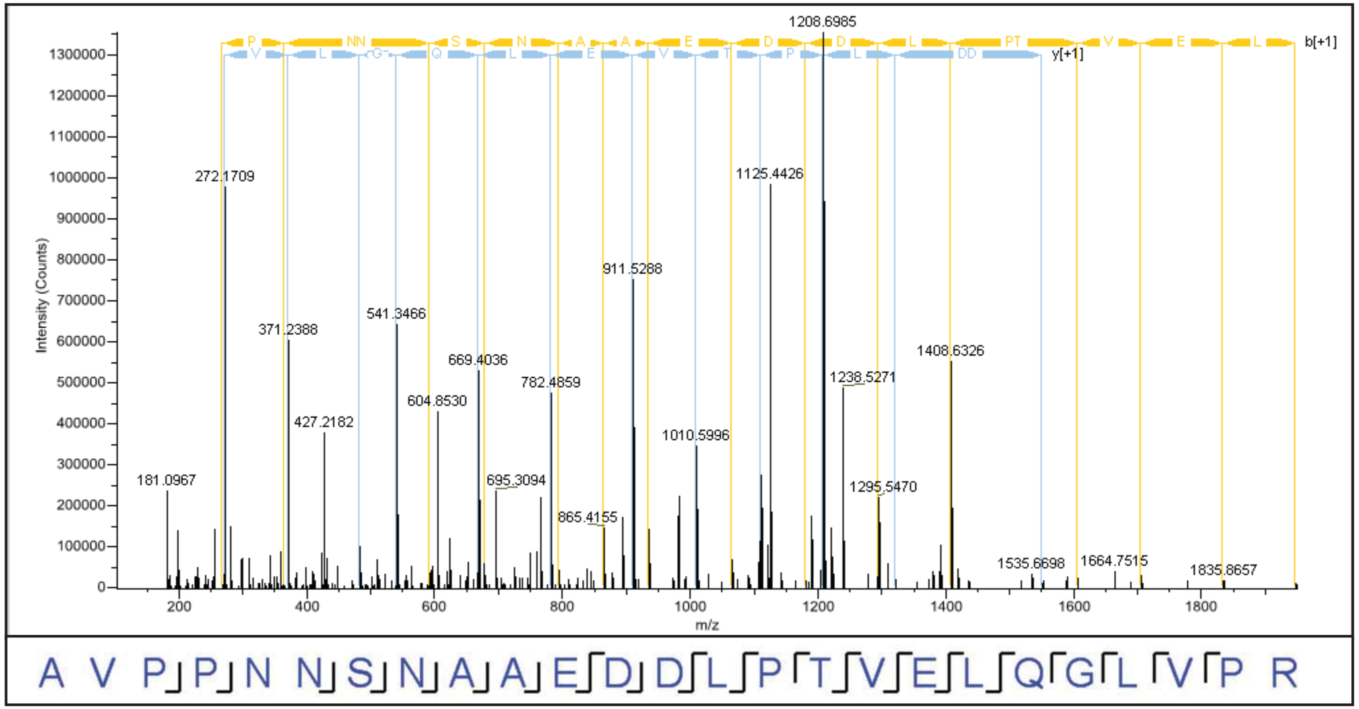

#1	b⁺	b²⁺	b³⁺	Seq.	y⁺	y²⁺	y³⁺	#2
1	72.04440	36.52584	24.68632	A				25
2	171.11282	86.06005	57.70912	V	2545.28940	1273.14834	849.10132	24
3	268.16559	134.58643	90.06005	P	2446.22098	1223.61413	816.07851	23
4	365.21836	183.11282	122.41097	P	2349.16821	1175.08774	783.72759	22
5	479.26129	240.13428	160.42528	N	2252.11544	1126.56136	751.37666	21
6	593.30422	297.15575	198.43959	N	2138.07251	1069.53989	713.36235	20
7	680.33625	340.67176	227.45027	S	2024.02958	1012.51843	675.34804	19
8	794.37918	397.69323	265.46458	N	1936.99755	969.00241	646.33737	18
9	865.41630	433.21179	289.14362	A	1822.95462	911.98095	608.32306	17
10	936.45342	468.73035	312.82266	A	1751.91750	876.46239	584.64402	16
11	1065.49602	533.25165	355.83686	E	1680.88038	840.94383	560.96498	15
12	1180.52297	590.76512	394.17917	D	1551.83778	776.42253	517.95078	14
13	1295.54992	648.27860	432.52149	D	1436.81083	718.90905	479.60846	13
14	1408.63399	704.82063	470.21618	L	1321.78388	661.39558	441.26614	12
15	1505.68676	753.34702	502.56710	P	1208.69981	604.85354	403.57145	11
16	1606.73444	803.87086	536.24966	T	1111.64704	556.32716	371.22053	10
17	1705.80286	853.40507	569.27247	V	1010.59936	505.80332	337.53797	9
18	1834.84546	917.92637	612.28667	E	911.53094	456.26911	304.51516	8
19	1947.92953	974.46840	649.98136	L	782.48834	391.74781	261.50096	7
20	2075.98811	1038.49769	692.66755	Q	669.40427	335.20577	223.80627	6
21	2133.00958	1067.00843	711.67471	G	541.34569	271.17648	181.12008	5
22	2246.09365	1123.55046	749.36940	L	484.32422	242.66575	162.11292	4
23	2345.16207	1173.08467	782.39221	V	371.24015	186.12371	124.41823	3
24	2442.21484	1221.61106	814.74313	P	272.17173	136.58950	91.39543	2
25				R	175.11896	88.06312	59.04450	1
