# Supplementary material for: Activation peptide of the coagulation factor XIII (AP-F13A1) as a new biomarker for the screening of colorectal cancer
Source: Clin Proteomics. 2018 Apr 9;15:15. doi: 10.1186/s12014-018-9191-3 (PMC5890357; doi:10.1186/s12014-018-9191-3)
Supplement: Supplementary file 6 — Additional file 6. Coefficients of Variation (CVs) of the ratios light/heavy. [file 12014_2018_9191_MOESM6_ESM.pdf]

Additional file 6
Coefficients of Variation (CVs) of the ratios light/heavy

CVs of the light/heavy peptides ratios calculated for each points of the calibration curve (Panel A), 120 injections across 40 biological replicates from the biobank 1 (Panel B) and 219 injections across 73 biological replicates from the biobanK 2 (Panel C).

Panel A - CVs of the light/heavy ratios calculated for AVPPNNSNAAEDDLPTVELQGVVPR peptide and AVPPNNSNAAEDDLPTVELQGLVPR peptide used for the establishment of the standard concentration curves in the serum (**a, b**).

Panel B - CVs of the light/heavy ratios calculated for AVPPNNSNAAEDDLPTVELQGVVPR peptide and AVPPNNSNAAEDDLPTVELQGLVPR peptide for individual patients of the biobank 1 (**a, b**). Distribution of the CVs of the light/heavy ratios for the two peptides over 40 biological replicates (**c**).

Panel C - CVs of the light/heavy ratios calculated for AVPPNNSNAAEDDLPTVELQGVVPR peptide and AVPPNNSNAAEDDLPTVELQGLVPR peptide for individual patients of the biobank 2 (**a, b**). Distribution of the CVs of the light/heavy ratios for the two peptides over 73 biological replicates (**c**).

Panel A

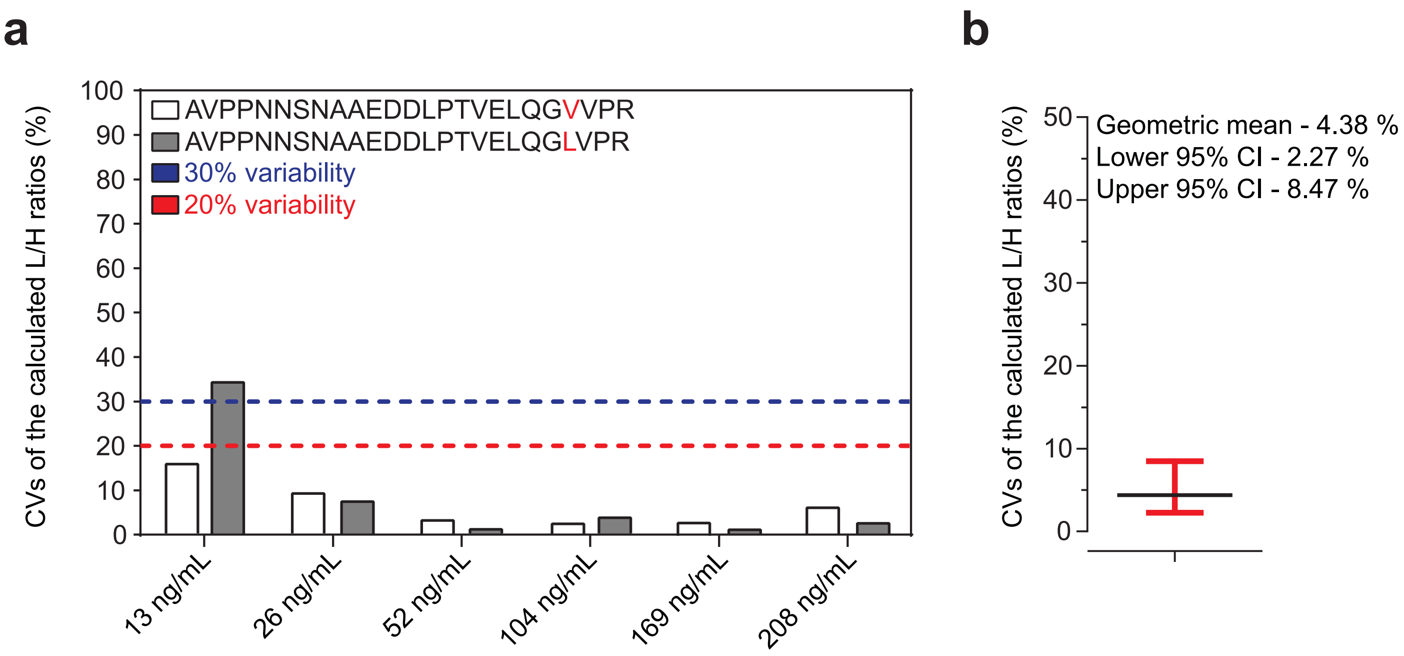

Panel B

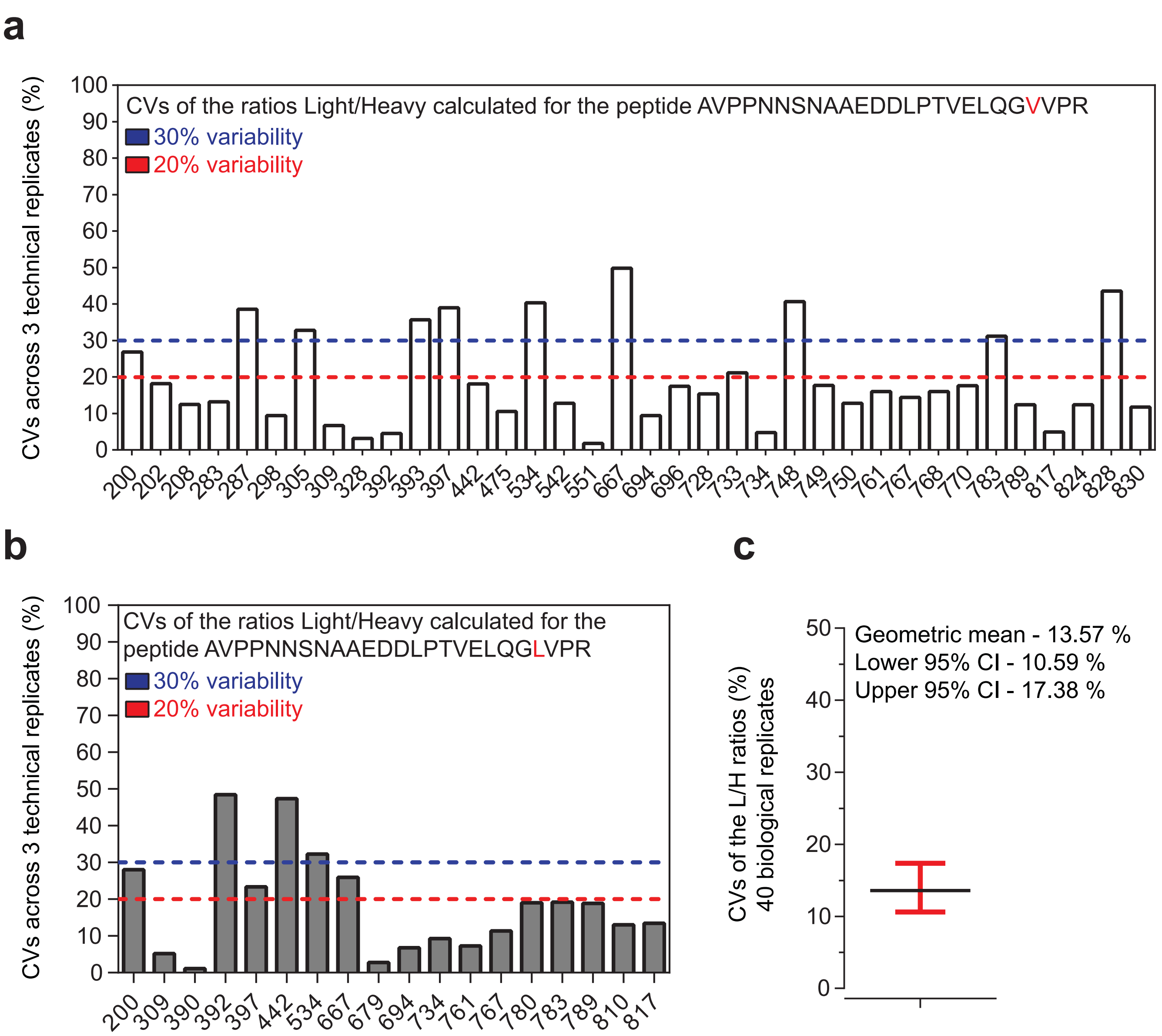

Panel C

a

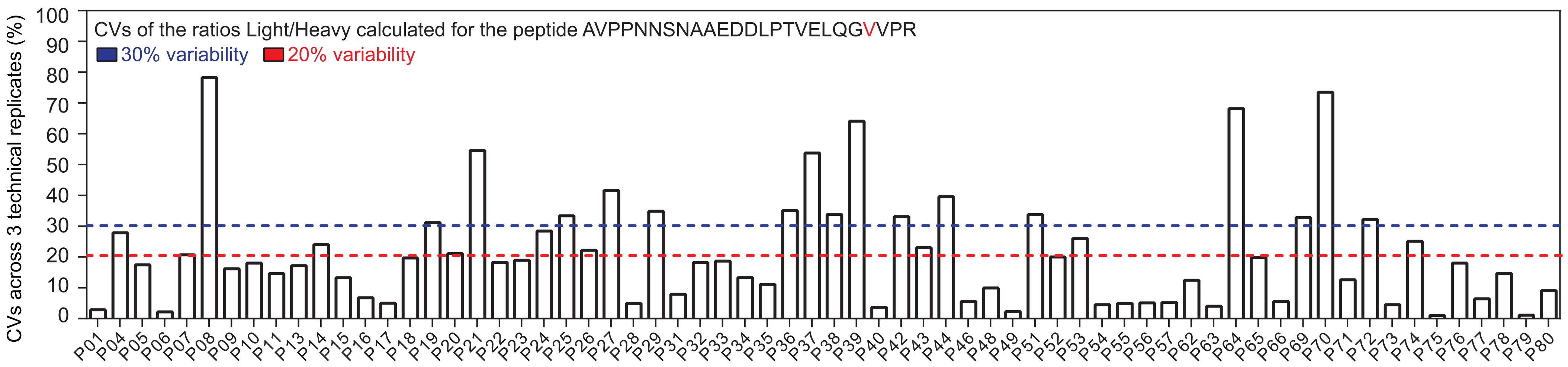

CVs of the ratios Light/Heavy calculated for the peptide AVPPNNSNAAEDDLPTVELQGVVPR
30% variability
20% variability
CVs across 3 technical replicates (%)
0
10
20
30
40
50
60
70
80
90
100
P01 P04 P05 P06 P07 P08 P09 P10 P11 P13 P14 P15 P16 P17 P18 P19 P20 P21 P22 P23 P24 P25 P26 P27 P28 P29 P31 P32 P33 P34 P35 P36 P37 P38 P39 P40 P42 P43 P44 P46 P48 P49 P51 P52 P53 P54 P55 P56 P57 P62 P63 P64 P65 P66 P69 P70 P71 P72 P73 P74 P75 P76 P77 P78 P79 P80

b

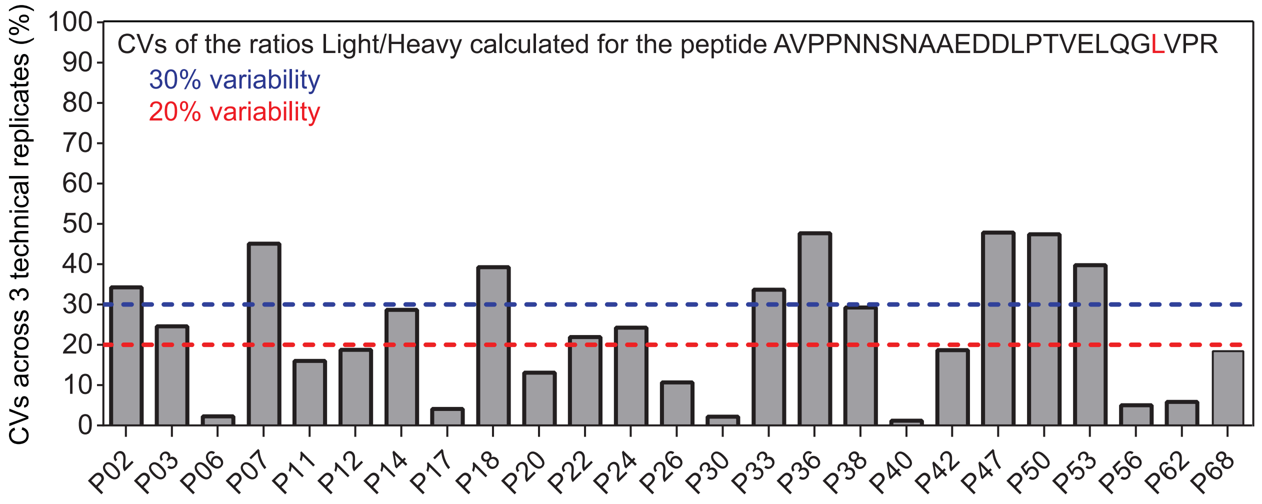

CVs of the ratios Light/Heavy calculated for the peptide AVPPNNSNAAEDDLPTVELQGLVPR
30% variability
20% variability
CVs across 3 technical replicates (%)
0
10
20
30
40
50
60
70
80
90
100
P02 P03 P06 P07 P11 P12 P14 P17 P18 P20 P22 P24 P26 P30 P33 P36 P38 P40 P42 P47 P50 P53 P56 P62 P68

c

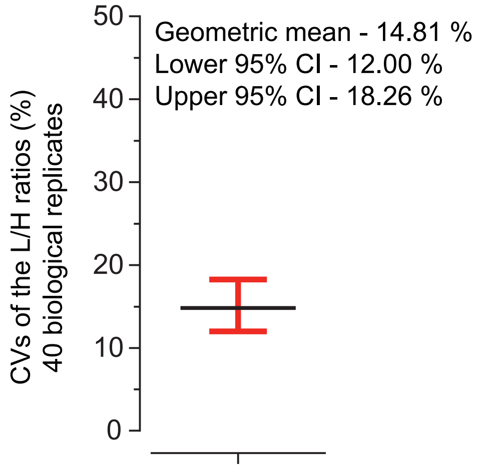

Geometric mean - 14.81 %
Lower 95% CI - 12.00 %
Upper 95% CI - 18.26 %
CVs of the L/H ratios (%)
40 biological replicates
0
10
20
30
40
50
