# Supplementary material for: Activation peptide of the coagulation factor XIII (AP-F13A1) as a new biomarker for the screening of colorectal cancer
Source: Clin Proteomics. 2018 Apr 9;15:15. doi: 10.1186/s12014-018-9191-3 (PMC5890357; doi:10.1186/s12014-018-9191-3)
Supplement: Supplementary file 7 — Additional file 7. Table summary of patients selected for the absolute quantification of the two isoforms of tAP-F13A1 by LC-PRM. [file 12014_2018_9191_MOESM7_ESM.pdf]

Supplementary table 2
Table summary of patients selected for the absolute quantification of the two isoforms of tAP-F13A1 by LC-PRM

Calculated concentration of tAP-F13A1 of individual healthy patients and patients. (**a**) Table of calculated concentration of the 40 patients extracted from the first biological biobank. (**b**) Table of calculated concentrations of the 73 patients extracted from the second biological biobank. The term “-” indicates that one of the tAP-F13A1 isoform is not detected during mass spectrometry analysis and analysis in the skyline software.

a

Replicate Name	Classification	Experimental concentration of AVPPNNSNAAEDDLPTVELQGVVPR (ng/mL)	Experimental concentration of AVPPNNSNAAEDDLPTVELQGLVPR (ng/mL)	Sum of 2 variants (ng/mL)
**283**	Control	110.4	-	110.4
**287**	Control	164.3	-	164.3
**305**	Control	256.9	-	256.9
**309**	Control	59.3	43.0	102.3
**390**	Control	-	230.6	230.6
**392**	Control	95.5	42.2	137.8
**393**	Control	223.9	-	223.9
**397**	Control	119.2	44.7	163.9
**442**	Control	191.8	47.4	239.2
**475**	Control	222.3	-	222.3
**534**	Control	144.3	73.0	217.3
**542**	Control	370.2	-	370.2
**679**	Control	-	88.6	88.6
**696**	Control	189.6	-	189.6
**728**	Control	203.4	-	203.4
**733**	Control	137.5	-	137.5
**748**	Control	61.4	-	61.4
**749**	Control	171.8	-	171.8
**750**	Control	220.2	-	220.2
**761**	Control	83.6	42.0	125.6
**810**	Control	93.9	-	93.9
**200**	Cancer	44.3	14.8	59.1
**202**	Cancer	129.1	-	129.1
**208**	Cancer	31.0	-	31.0
**298**	Cancer	86.5	46.4	132.9
**328**	Cancer	50.7	-	50.7
**551**	Cancer	51.3	-	51.3
**667**	Cancer	37.3	-	37.3
**694**	Cancer	32.1	14.2	46.2
**734**	Cancer	88.7	-	88.7
**767**	Cancer	57.0	32.3	89.3
**768**	Cancer	53.2	25.3	78.5
**770**	Cancer	21.7	-	21.7
**780**	Cancer	-	42.8	42.8
**783**	Cancer	33.7	18.0	51.8
**789**	Cancer	63.2	-	63.2
**817**	Cancer	25.9	11.4	37.3
**824**	Cancer	17.0	-	17.0
**828**	Cancer	46.4	-	46.4
**830**	Cancer	-	19.9	19.9

b

Replicate Name	Classification	Experimental concentration of AVPPNNSNAAEDDLPTVELQGVVPR (ng/mL)	Experimental concentration of AVPPNNSNAAEDDLPTVELQGLVPR (ng/mL)	Sum of 2 variants (ng/mL)
**P01 - VAL370**	Control	148.7		148.693
**P02 - VAL373**	Control		102.7	102.680
**P03 - VAL378**	Control		110.9	110.919
**P04 - VAL380**	Control	137.5		137.455
**P05 - VAL400**	Control	148.1		148.126
**P09 - VAL413**	Control	197.4		197.425
**P10 - VAL414**	Control	465.1		465.147
**P11 - VAL423**	Control	89.8	38.9	128.720
**P012 - VAL424**	Control		153.6	153.645
**P013 - VAL425**	Control	103.3		103.315
**P014 - VAL426**	Control	48.6	206.9	255.461
**P015 - VAL427**	Control	153.8		153.816
**P016 - VAL433**	Control	144.1		144.137
**P017 - VAL435**	Control	90.1	99.1	189.169
**P18 - VAL437**	Control	47.8	48.3	96.111
**P19 - VAL444**	Control	174.4		174.412
**P20 - VAL448**	Control	110.4	82.4	192.786
**P21 - VAL488**	Control	262.8		262.776
**P22 - VAL497**	Control	111.5	133.1	244.641
**P23 - VAL498**	Control	230.3		230.324
**P25 - VAL505**	Control	205.2		205.185
**P26 - VAL511**	Control	77.5	62.5	140.065
**P27 - VAL512**	Control	245.6		245.594
**P28 - VAL514**	Control	187.7		187.750
**P42 - VAL010**	Control	70.1	74.3	144.433
**P43 - VAL100**	Control	322.4		322.373
**P44 - VAL105**	Control	432.4		432.415
**P47 - VAL116**	Control		145.3	145.328
**P48 - VAL117**	Control	137.1		137.051
**P49 - VAL119**	Control	476.9		476.866
**P50 - VAL121**	Control		53.9	53.933
**P51 - VAL122**	Control	153.6		153.606
**P52 - VAL127**	Control	159.4		159.399
**P53 - VAL129**	Control	39.0	25.1	64.071
**P54 - VAL133**	Control	106.0		105.981
**P55 - VAL135**	Control	231.9		231.926
**P56 - VAL136**	Control	164.4	80.4	244.849
**P57 - VAL141**	Control	195.7		195.737
**P62 - VAL150**	Control	161.7	118.0	279.733
**P63 - VAL151**	Control	199.5		199.521
**P75 - VAL350**	Control	164.1		164.109
**P76 - VAL352**	Control	178.6		178.620
**P77 - VAL353**	Control	99.0		99.037
**P78 - VAL355**	Control	100.4		100.416
**P79 - VAL357**	Control	149.0		149.041
**P80 - VAL362**	Control	156.2		156.199
**P31 - VAL569**	Cancer	106.8		106.845
**P65 - VAL204**	Cancer	60.1		60.127
**P66 - VAL233**	Cancer	67.5		67.491
**P69 - VAL260**	Cancer	33.4		33.424
**P07 - VAL409**	Cancer	47.7	18.1	65.772
**P24 - VAL503**	Cancer	71.9	50.2	122.062
**P29 - VAL525**	Cancer	95.3		95.276
**P30 - VAL556**	Cancer		132.9	132.904
**P34 - VAL613**	Cancer	107.3		107.347
**P35 - VAL643**	Cancer	38.7		38.745
**P36 - VAL662**	Cancer	54.5	36.8	91.300
**P71 - VAL341**	Cancer	28.4		28.421
**P72 - VAL342**	Cancer	29.4		29.362
**P74 - VAL345**	Cancer	39.3		39.260
**P06 - VAL408**	Cancer	34.1	14.5	48.691
**P32 - VAL600**	Cancer	112.9		112.947
**P33 - VAL607**	Cancer	53.2	18.3	71.442
**P37 - VAL666**	Cancer	75.0		75.015
**P39 - VAL006**	Cancer	28.5		28.542
**P40 - VAL037**	Cancer	75.8	47.3	123.090
**P46 - VAL114**	Cancer	118.1		118.053
**P08 - VAL410**	Cancer	35.7		35.675
**P38 - VAL667**	Cancer	52.0	87.7	139.676
**P64 - VAL178**	Cancer	37.3		37.263
**P68 - VAL254**	Cancer		22.1	22.120
**P70 - VAL336**	Cancer	27.7		27.704
**P73 - VAL344**	Cancer	43.5		43.473
